# BHi-Cect: a top-down algorithm for identifying the multi-scale hierarchical structure of chromosomes

**DOI:** 10.1093/nar/gkaa004

**Published:** 2020-02-03

**Authors:** Vipin Kumar, Simon Leclerc, Yuichi Taniguchi

**Affiliations:** 1 Laboratory for Cell Systems Control, RIKEN Center for Biosystems Dynamics Research, Suita, Osaka 5650874, Japan; 2 PRESTO, Japan Science and Technology Agency, Kawaguchi, Saitama 3320012, Japan

## Abstract

High-throughput chromosome conformation capture (Hi-C) technology enables the investigation of genome-wide interactions among chromosome loci. Current algorithms focus on topologically associating domains (TADs), that are contiguous clusters along the genome coordinate, to describe the hierarchical structure of chromosomes. However, high resolution Hi-C displays a variety of interaction patterns beyond what current TAD detection methods can capture. Here, we present BHi-Cect, a novel top-down algorithm that finds clusters by considering every locus with no assumption of genomic contiguity using spectral clustering. Our results reveal that the hierarchical structure of chromosome is organized as ‘enclaves’, which are complex interwoven clusters at both local and global scales. We show that the nesting of local clusters within global clusters characterizing enclaves, is associated with the epigenomic activity found on the underlying DNA. Furthermore, we show that the hierarchical nesting that links different enclaves integrates their respective function. BHi-Cect provides means to uncover the general principles guiding chromatin architecture.

## INTRODUCTION

Chromosome conformation is tightly linked to DNA function and activity as illustrated by heterochromatin condensation, enhancer-promoter looping or gene regulatory region accessibility (1). It is only until recently that we were able to probe such relations at a genome-wide scale with the emergence of chromosome conformation capture technologies, notably their high-throughput version coined Hi-C (2). Hi-C, through deep sequencing of DNA fragments ligating proximal genomic DNA, yields a genome-wide inventory of physical interactions between every accessible locus, capturing the actual folding of the chromatin inside the nucleus.

It is widely documented that chromosomes have the tendency to form insulated structures, that are believed to provide dynamic ‘microenvironments’ to fine-tune the activity of the underlying DNA regions (3). This notion motivated studies to apply various clustering methods on Hi-C data. One of the main insights these studies revealed is the existence of recurring insulated clusters at small and large scales displaying characteristic biological features. At small scales, topologically associating domains (TADs) were identified as megabase sized self-interacting contiguous chromosome regions (4). TADs are considered as fundamental units of chromosome organisation because of their segmentation of the chromosome into functionally cohesive regions (4–7). At large scales, compartments (8) were identified as a broad segmentation of the entire genome into active and inactive regions (8). Furthermore, at a more general level, modeling analyses of Hi-C data indicated that the multi-scale hierarchical organisation of chromosomes is based on successive nesting of loci-clusters following a fractal globule conformation (8,9).

Eventually, a key goal would be to characterize how this hierarchical organisation of insulated chromosome clusters bridges whole chromosomes with individual loci. In this effort, TADs are widely used as a fundamental brick, whose aggregation eventually forms the whole chromosome in a bottom-up fashion (10–15). However, TADs postulate clustering among neighboring loci that are contiguous along the genome coordinate, which can mask more complex interaction patterns often seen in more recent high resolution Hi-C data (16). These complex patterns often result from important processes such as some chromatin looping, chromatin allosteric effect or sub-TADs formation (17).

To overcome this limitation, we propose a new clustering method termed BHi-Cect. BHi-Cect clusters loci by considering all possible combinations without imposing contiguity along the genome coordinate using spectral clustering (18), thus circumventing the limitations of current TAD-based methods. Furthermore, the recursive design of BHi-Cect enables us to find further embedded sub-clusters, revealing the nested organization of loci clusters at all scales of the chromosome hierarchy. The results from this novel top-down approach provide a description of chromosome architecture linking chromosomal hierarchy with the underlying DNA function and activity.

## MATERIALS AND METHODS

### Clustering algorithm

BHi-Cect first formats the sequenced paired-end reads yielded by Hi-C methods (2) into a Hi-C contact matrix *W*(*i*, *j*), where each entry stands for the total read number, or the interaction frequency, between loci *i* and *j*. Here, loci refer to the equally-sized and non-overlapping bins segmenting the genome at the resolution of the considered Hi-C dataset. The matrix is then normalized using the Knight–Ruiz (KR) matrix balancing normalization (19) so as to compensate bias in enzymatic digestion frequencies at every locus. All Hi-C data were downloaded and processed into KR normalized interaction matrices using Juicer tools (20). Furthermore, the matrix is scaled with a Box–Cox transformation optimizing for normality to mitigate the dispersion of Hi-C interaction frequencies (21). We used a specific Box–Cox lambda for each chromosome using the MASS R package (22), which implements the maximum likelihood estimate procedure described in (23).

The chromatin hierarchy is captured by recursively bisecting genomic loci sets starting from the whole chromosome. For the bisection, we use the principle of spectral clustering (24) with some modifications. In this method, the chromatin interactions are considered as a network, where each vertex corresponds to a chromosome locus and edge weights between vertices is set by their normalized interaction frequency. The bisection is performed so as to maximize the total sum of edge weights within the bisected loci groups through spectral clustering (18). To compute this, we first derive the degree matrix, *D*, which is a diagonal matrix whose entries correspond to the sum of all the interaction weights for the considered loci. We then derive the Laplacian matrix, *L*, from the equation:

$$\begin{equation*}{{L\ }} = {{\ I}} - {D^{ - 1}}{{W}}\end{equation*}$$

with *I* being the identity matrix of the same size as the Hi-C matrix *W*. Here, we use the random walk normalized formulation of the Laplacian matrix, which corrects for undesirable topological biases, notably regarding highly interacting loci, while maintaining statistical stability (25). Spectral clustering then requires the eigendecomposition of this Laplacian matrix to derive the second smallest eigenvalue and associated eigenvector, also known as the Fiedler vector. Finally, we perform a *k*-means clustering (*k* = 2) on this vector, for which we collect the consensus from five different seedings, in order to find the optimal bisection (24). This bisection is repeated until we only have a 2 × 2 matrix.

**Figure 1.**
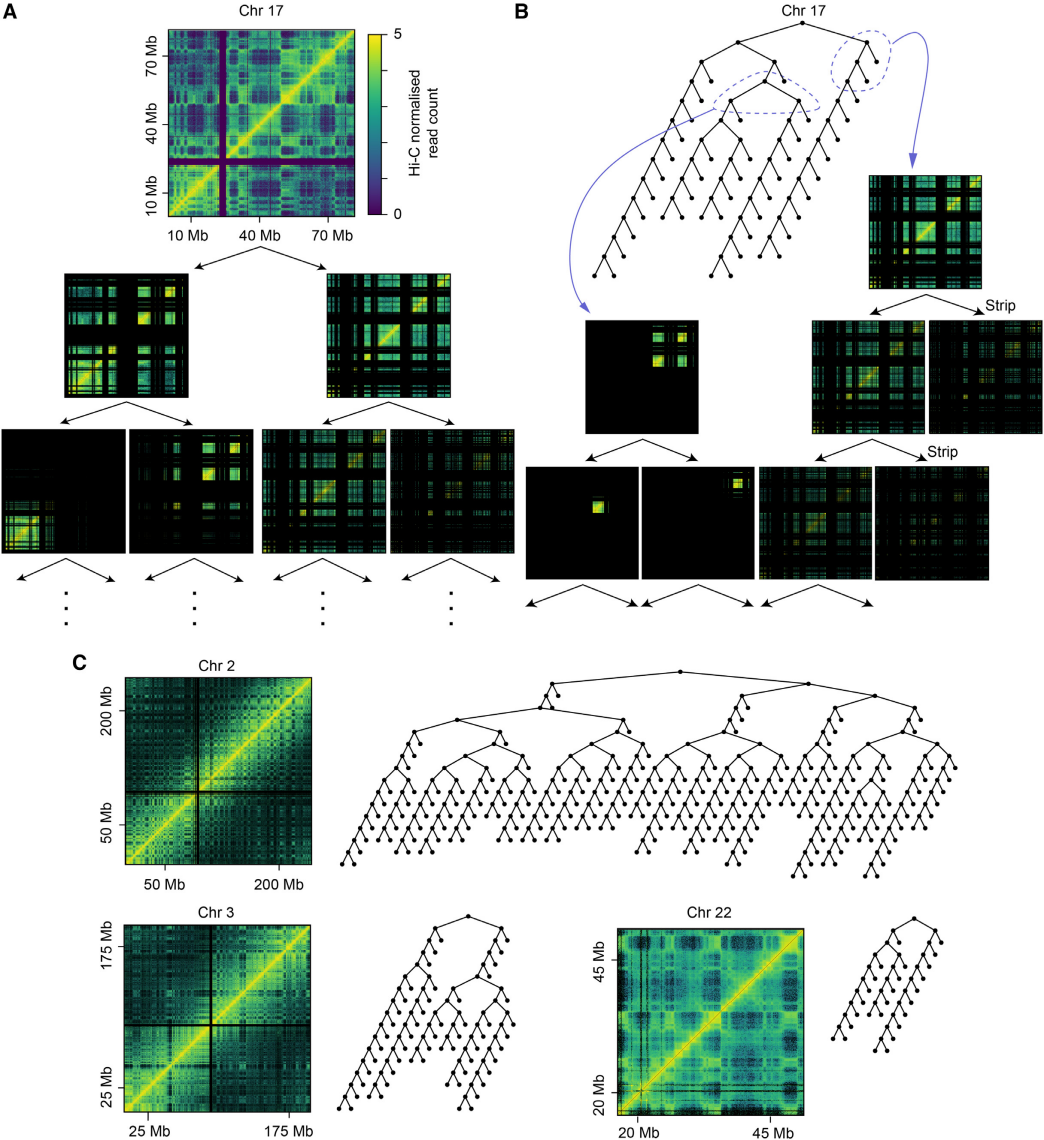
Clusters found by BHi-Cect and their representation as a tree. (**A**) Example of successive bissections performed by BHi-Cect illustrated with Hi-C contact heatmap showing interaction frequencies between loci. Deep purple regions in the original Hi-C heatmap (top) correspond to unmapped regions. (**B**) BHi-Cect partition tree (BPT) representing the hierarchical relationships among the outputted loci clusters (top). Heatmap representations for split (bottom left) and strip (bottom right) partitions are also shown. (**C**) Diversity of BPT topology observed across different chromosomes.

For each bisection, we further evaluated the self-interaction level of the resulting partitions using the expansion metric (26). The expansion metric is defined as the ratio dividing the sum of edge weights crossing the considered partition over the sum of edge weights inside the considered partition. The mathematical formulation for the expansion metric for a particular partition *C*_1_ is given by:

$$\begin{equation*}\ {{\rm expansion}_{{c_1}}} = \frac{{\mathop \sum \nolimits_{x \in {C_1}} \mathop \sum \nolimits_{y \in {C_2}} w\left( {x,y} \right)}}{{\mathop \sum \nolimits_{x \in {C_1}} \mathop \sum \nolimits_{y \in {C_1}} w\left( {x,y} \right)}}\end{equation*}$$

where *x* and *y* represent individual chromosome loci, and *w*(*x*, *y*) represents the edge weight between loci *x* and *y*. *C*_1_ is the considered partition and *C*_2_ is the other partition resulting from the same bisection. We consider a partition well insulated when the expansion metric is smaller than 1, meaning a majority of edge weights exist within the considered partition. We named a bisection ‘split’ or ‘strip’ depending on whether it yielded two or one insulated partitions respectively. If neither partitions displayed an expansion value smaller than one, we stopped the algorithm. To summarize the result, we built a tree structure called a BHi-Cect partition tree (BPT) by aggregating the found partitions (Figure 1A). To validate the scalability of BHi-Cect we benchmarked it against Hi-C datasets of different resolutions (Supplementary Figure S12). The code for the algorithm is available at https://github.com/princeps091-binf/BHi-Cect.

### Definition of top and bottom enclaves and BPT leaves

We observed that all BPTs accumulated split branching at the top and then tended to have long streaks of strip branching toward their deeper end (Supplementary Figure S5). These features can be captured through three characteristic BPT nodes or enclaves. Firstly top enclaves correspond to the all BPT nodes yielding a strip branching but above which we only find split branching events (first strips). Secondly bottom enclaves are defined as all BPT nodes below which we only find strip branching events and directly resulting from a split branching event (last splits). Thirdly leaves correspond to tree nodes marking the end of the BPT, meaning they don’t have any children nodes despite having an expansion metric smaller than 1 or they contain less than two loci.

### Definition of nestedness

Nestedness (NS) allows a systematic exploration of the obtained BPT that is not affected by the topological differences observed between chromosomes. To do so, we assign the same value for partitions, which because of their BPT positions, are shared across all chromosomes. We refer to these topologically invariant partitions as top enclaves, bottom enclaves and BPT leaves as described above. We set the nestedness value of these partitions to 0 for top enclaves, 0.5 for bottom enclaves and 1 for leaf partitions. We then linearly interpolate nestedness values for any intermediary partitions. To achieve this, we consider partitions present below and above bottom enclaves partition separately.

For partitions between top and bottom enclaves, nestedness is defined as:

$$\begin{equation*}{{\rm NS}_i} = \frac{{0.5*{D_i} - {\rm min}\left( {{D_i}} \right)}}{{\left( {{\rm max}\left( {{D_i}} \right) - {\rm min}\left( {{D_i}} \right)} \right) + 1}}\end{equation*}$$

Here, *D_i_*, or root distance, is the number of branches needed to link the BPT root with the considered partition. min(*D*_i_) corresponds to the root distance for the closest top enclave. max(*D*_i_) corresponds to the root distance for the furthest bottom enclave directly descending from the considered partition. Adding 1 to the denominator ensures that the obtained nestedness values will be strictly smaller than 0.5.

For partitions between bottom enclaves and leaf partitions, nestedness is defined as:

$$\begin{equation*}{{\rm NS}_i} = \frac{{0.5*{D_i} - {\rm min}\left( {{D_i}} \right)}}{{{\rm max}\left( {{D_i}} \right) - {\rm min}\left( {{D_i}} \right)}}\ + 0.5\end{equation*}$$

Here, min(*D*_i_) corresponds to the root distance for the closest bottom enclave. max(*D*_i_) corresponds to the BPT leaf directly descending from the considered partition.

### Variation of information

To assess the agreement in clustering results between different datasets, we used the variation of information (VI) (27). This value is based on information theory and can give us a measure of the hypothetical distance regarding the agreement between different loci clustering results. The VI between two loci clustering results, *C* and *C’*, is given by:

$$\begin{equation*}VI \left( {C,C^{\prime}} \right) = H\left( C \right) + H\left( {C^{\prime}} \right) - 2I\left( {C,C^{\prime}} \right)\end{equation*}$$

where *H*(*C*) represents the entropy of *C*, and *I*(*C*,*C′*) represents the mutual information between *C* and *C*′. Assuming that *C* and *C*′ contains *K* and *K*′ clusters respectively, these values are given by:

$$\begin{equation*}H \left( C \right) = - \mathop \sum \nolimits_{k = 1}^K P\left( k \right)\log\\P\left( k \right)\end{equation*}$$

$$\begin{equation*}I \left( {C,C^{\prime}} \right) = \mathop \sum \nolimits_{k = 1}^K \mathop \sum \nolimits_{k^{\prime} = 1}^{K^{\prime}} P\left( {k,k^{\prime}} \right)\ \log\frac{{P\left( {k,k^{\prime}} \right)}}{{P\left( k \right)P\left( {k^{\prime}} \right)}}\end{equation*}$$

with *P*(*k*) representing the probability of loci being identified as part of the *k*th cluster in *C*, and *P*(*k*, *k*′) representing the joint probability of loci being simultaneously part of the *k*th and *k′*th cluster in *C* and *C’*, respectively. Thus *VI*(*C,C′*) will be high when the entropy *H* of the compared clusterings is high, meaning they contain many clusters respectively or their mutual information *I* is low, meaning the compared clusterings have very little in common in terms of how they group loci. Conversely, *VI*(*C,C′*) will be low when the compared clusterings contain few clusters respectively or the compared clusterings group loci in a very similar manner.

To examine the statistical significance of the agreement between our enclave clustering and a reference clustering (e.g. TADs), we derived an empirical *P*-value based on this VI. To compute it, we first randomly generated 500 sets of chromosome clustering. This random clustering comes from a recursive bisection of the genome into clusters that match the size and number of loci in the original partition tree, but are otherwise chosen at random. We then calculated the VI for each random clustering against the reference clustering. Then we counted the number of random clustering that had a smaller VI than the one between our enclave clustering and the reference clustering (*r*). We then derived the empirical *P*-value using the following formula:

$$\begin{equation*}{\rm empirical}\ P-{\rm value}\ = \frac{{r + 1}}{{n + 1}}\end{equation*}$$

with *n* equal to the total number of clustering considered (*n* = 501).

### Statistical analyses of genome-wide data

#### DNA loops integration

We downloaded the genome coordinates of the DNA loop anchor sites found by (16) using the HICCUPS method on the IMR90 Hi-C data (5 kb) (16). For each DNA loop, we then computed the overlap of each member of the anchor site pair with bottom enclaves derived from the same Hi-C data. We then examined for each DNA loop whether both anchor sites were present in the same bottom enclave. Finally we reported the percentage of DNA loops with both anchor sites in the same bottom enclave. For the comparison with TADs we repeated the analysis but using TADs instead of bottom enclaves.

#### Summarizing the overlap between BHi-Cect clustering and epigenomic data

To compare our clustering results with epigenomic features, we used genome-wide BED datasets from public databases. All epigenomic data (ChIP, RNA-seq, CAGE, LAD, DNase, ChromHMM) for all cell lines were downloaded from the NIH Roadmap Epigenomics Mapping Consortium database website (28) and ENCODE project website (29).

We first find the overlaps between the epigenomic feature of interest and the considered cluster. We then compute one of three summary statistics for these overlaps:

*Size of the overlap*

$$\begin{equation*}\overline {{x_s}} = \frac{{\mathop \sum \nolimits_{i = 1}^n b{p_i}\mathop \cap \nolimits^ cl}}{{b{p_{cl}}}}\end{equation*}$$

with *i* representing a particular site for the considered epigenomic feature, *n* representing the total number of sites for the considered epigenomic feature along the genome, *bp_i_* representing the number of basepairs for site *i*, *cl* representing the considered cluster and *bp_cl_* representing the number of basepairs composing the considered cluster. The metric essentially computes the proportion of the cluster overlapped by the considered epigenomic feature.

*Average overlapping signal intensity per bp*

$$\begin{equation*}\overline {{x_I}} = \frac{{\mathop \sum \nolimits_{i = 1}^n \left( {b{p_i}\mathop \cap \nolimits^ cl} \right) \times {I_i}}}{{b{p_{cl}}}}\end{equation*}$$

with *i* representing a particular site for the considered epigenomic feature, *n* representing the total number of sites along the genome for the considered epigenomic feature, *bp_i_* representing the number of basepairs for site *i*, *I_i_* representing the intensity reported for site *i*, *cl* representing the considered cluster and *bp_cl_* representing the number of basepairs composing the considered cluster.

*Number of overlaps*

$$\begin{equation*} \overline {{x_n}} = \mathop \sum \limits_{i = 1}^n {\delta _i}\ \left\{ {\begin{array}{@{}*{1}{c}@{}} {0\,\,\, {\rm if}\,\,\, {{\rm peak}_i}\mathop \cap \nolimits^ cl = 0}\\ {1\,\,\, {\rm if}\,\,\, {{\rm peak}_i}\mathop \cap \nolimits^ cl \ne 0} \end{array}} \right. \end{equation*}$$

with *i* representing a particular site for the considered epigenomic feature, *n* representing the total number of sites along the genome for the considered epigenomic feature, *δ*_*i*_ representing the overlap counter and *cl* representing the considered cluster.

#### Correspondence analysis

We performed a correspondence analysis (CA) (30) of bottom enclaves with respect to their TF content. Briefly with the IMR90 cell line ChIP-seq data for Ccaat-enhancer-binding proteins (CEBP), RCOR1, MAZ, MXI, RFX5, MafK, FOS, USF2, NFE2L2 and ELK1 downloaded from the ENCODE website (28), we build a contingency table summarizing the number of binding sites for every TF found in each bottom enclave. More specifically we computed in each bottom enclave the number of overlaps (as defined above) for every TF. We then applied CA onto this contingency table and extracted the first two dimensions for both the row (bottom enclave) and column (TF) space.

#### Gene set enrichment analysis gene ranking

For this analysis when we rank genes based on the RNAP2 (IMR90) or H3K4me3 (K562) content of bottom enclaves, we use the respective narrowpeak bed files of these epigenomic features from the corresponding cell line. We then computed the average overlapping signal intensity per bp (as described above) to summarize the bottom enclave content for these different epigenomic features.

#### Feature enrichment analysis

In the feature enrichment analysis the ‘clusters’ considered are actually individual loci. More specifically for each locus, we compute the number of overlaps with the considered epigenomic feature (as defined above). We then assigned to each locus the nestedness value corresponding to the most nested partition they could be found in. Because we apply the same measure of nestedness across every chromosome, we can now examine the genome-wide relation of nestedness with various epigenomic features. Since we are interested in the relative change along nestedness, we further divided each locus overlap value by the average number of overlap observed across all the loci of the same top enclave.

$$\begin{equation*}\ {{\rm FE}_i} = \frac{{\overline {{x_i}} }}{{\overline {{{\rm top}_i}} }}\end{equation*}$$

where FE_i_ represents the feature enrichment value for a particular locus *i*, $\overline {{x_i}}$ represents the number of overlaps observed for the considered locus *i* and $\overline {{{\rm top}_i}}$represents average number of overlaps observed for all loci belonging to the same top enclave as the considered locus *i*.

This is why we talk of feature enrichment, since we are examining the relative enrichment in overlaps with loci as we progress from top enclaves all the way down to BPT leaves. We also performed the same analysis with the average overlapping signal intensity per bp and the overlap size metrics described above.

To test the significance of the difference in the number of overlaps observed among loci across different nestedness levels, we performed a Poisson ratio test between the expected genome-wide frequency of annotated regions for the epigenomic feature of interest and the observed frequency of overlap found within the considered set of loci.

### Analyses of functional similarity between bottom enclaves

For this analysis, we used two approaches to evaluate the functional similarity between pairs of bottom enclaves. Firstly, we considered bottom enclave functionality as summarized by their TF content. Using the first two dimensions derived from the CA applied on the contingency table summarizing the TF content of every bottom enclave we obtained a reduced 2D space (described earlier). Within it, we evaluated functional similarity by computing the pairwise euclidean distance for every combination of bottom enclaves in each chromosome.

Secondly we considered bottom enclave functionality as summarized by their significantly enriched GO-terms content. We then computed the semantic similarity (resnik similarity) for every pairwise combination of bottom enclaves in each chromosome with respect to their respective significantly enriched GO-terms.

The significance of the GO-term enrichment was empirically estimated for each bottom enclaves by considering only GO-terms with extreme fold enrichment (FE) values compared to the distribution of GO-terms FE values derived from randomly generated corresponding bottom enclaves. These random bottom enclaves come from a recursive bisection of the genome into clusters that match the size and number of loci in the original partition tree, but are otherwise chosen at random. The threshold used to identify ‘extreme’ FE values in the random FE distribution was set as:

$$\begin{equation*}{{\rm FE}_{{\rm significant}}} >2\ \times {\rm IQR} + 75{\rm th}\ {\rm percentile}\end{equation*}$$

with FE_significant_ representing the FE significance threshold and IQR representing the interquartile range of the considered distribution. A non-parametric threshold was used to account for the non-Gaussian distribution of random FE values.

This procedure gives us a set of enriched GO-terms for each bottom enclave which we can now use to evaluate the pairwise semantic similarity between bottom enclaves. We quantified the pairwise semantic similarity between the associated sets of enriched GO-terms using the resnik similarity metric (31). Briefly, the resnik similarity is based on the notion of information content of a GO-term defined as minus logarithm of the probability of finding the GO-term in the complete GO-annotation corpus.Thus, for the considered pair of GO-term sets, we compute the information content of the lowest common ancestor in the associated GO-tree (32).

Both TF-distance and semantic similarity were compared with corresponding pairwise chromosome coordinate distances between bottom enclaves. We used the average coordinate of all constitutive loci as reference coordinate respectively for each bottom enclave.

### Gene set enrichment analysis

We first determined the gene content of every bottom enclave and considered only transcriptionally active genes. We do this by computing all the overlapping RNA-seq peak sites and listing the associated gene ensembl ID annotation for every bottom enclave. We then ranked genes according to specific epigenomic aspects of the respective bottom enclaves containing them (as described earlier). The gene sets required for GSEA were formed based on the GO annotation of human genes provided by the GO consortium websites (32). Finally we computed the gene set enrichment and *P*-values as described by (33). To account for the multiple testing effect, we only used FDR corrected p-values.

## RESULTS

### Description of the method

BHi-Cect is a top-down algorithm for the characterization of the chromosome's hierarchical structure. This top-down analysis was inspired by established image segmentation methods in computer vision, which identify distinct objects through the recursive partition of the full image (18). We applied this approach to detect isolated chromatin loci clusters from the totality of chromosomal interactions yielded by Hi-C.Similarly to the computer vision approach, BHi-Cect recursively partitions the chromosome's loci into two clusters, starting from the whole chromosome working its way down to individual loci (Figure 1A). The partitioning was done using spectral clustering (24), which finds the two loci clusters sharing the least number of chromosomal interactions. The recursive partitioning was repeated until the resulting chromosome clusters had <2 loci. Eventually, the overall process forms a partition tree, which represents the nested hierarchical architecture of the chromatin structure (Figure 1A).

Using BHi-Cect, we analyzed Hi-C data from human IMR90 chromosomes, in which interactions among the chromosome's loci were analyzed at a 50 kb resolution (16). We observed that BHi-Cect could isolate non-contiguous self-interacting loci clusters as indicated by the plaid pattern regions in the heatmap representation of the associated Hi-C data (e.g. the first partitioning in Figure 1A). We confirmed that the isolated clusters were robustly detected when using datasets with different resolutions (5 kb–1 Mb), sparsity level, read depth, derived from different biological replicates and subjected to different normalisation schemes (Supplementary Figures S1, S2 and S4). Importantly, we noticed that BHi-Cect gave two kinds of bisection characterized by distinctive heatmap patterns derived from the resulting loci clusters. The first bisection kind, resulted in loci-clusters with similar interaction densities and covering different chromosome locations (Figure 1A, bottom left), indicating the presence of two strongly self-interacting chromosome-clusters nested within the original cluster. The second kind resulted in loci-clusters with interaction densities that were substantially higher in one compared to the other and covering deeply intersecting chromosome regions (Figure 1A, bottom right), indicating that the original cluster is composed of only one strongly self-interacting cluster with an associated auxiliary loci-cluster. These observations suggest that the clusters derived from our recursive partitioning display different hierarchical relations which can be efficiently represented with a tree structure whose branches will reflect the different nesting dynamics observed in the chromosome hierarchy.

Figure 1B shows an example of such a tree representation that captures the hierarchical relationships among the chromosome clusters, which we call the BHi-Cect partition tree (BPT). Here, the vertices in the BPT represent the chromosome clusters, and the branches from a vertex represent the existence of sub-clusters nested within the cluster. For convenience, we named branchings yielding one or two preferentially self-interacting chromosome clusters as strip and split branching respectively. By analyzing this BPT for every chromosome, we observed an extensive topological variety in terms of both tree depth and overall number of branching events (Figure 1C). This highlights the variety of chromosome architecture both in terms of number of clusters found and their complexity.

### Enclaves as fundamental structural units

**Figure 2.**
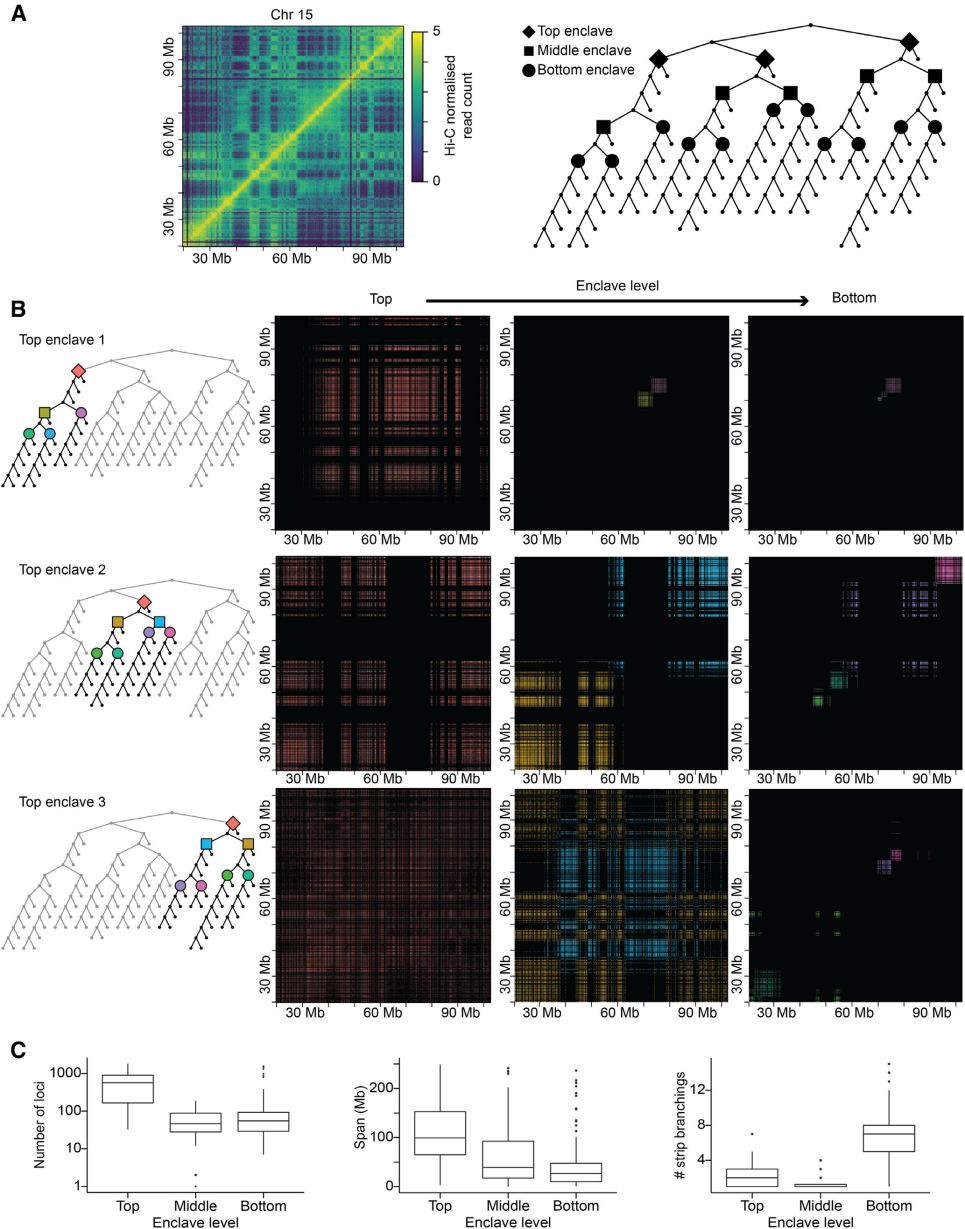
Enclaves are key positions in the BHi-Cect partition tree highlighting salient interaction patterns. (**A**) The first strip branching (Top) and following split branching (Mid and Bottom) events represent tree topological landmarks that highlight chromosome structures we call enclaves. (**B**) Examples of heatmap patterns in enclave regions at different levels. (**C**) Boxplots for the number of loci (left), span (middle) and number of strip branching (right) in enclaves at different hierarchical levels.

Next we examined heatmap patterns in the genomic regions highlighted by the insulated loci clusters detected at different branching points (Figure 2A and B). Since the detected clusters can be non-contiguous, we called them ‘enclaves’ to distinguish them from the more conventional contiguous clusters like TADs. Also, because enclaves have a parent-child relationship as described in the BPT, we classified them depending on their relative position in the BPT (Figure 2A). Enclaves at the top of the BPT generally exhibited either the plaid patterns or entirely covered the heatmap (left column heatmaps in Figure 2B). This indicated the presence of large-scale interwoven structures similar to compartments, and further suggested global interactions between loci across the whole chromosome. In terms of size, top enclaves spanned on average 120 Mb containing thousands of loci (Figure 2C). Enclaves lower in the BPT became smaller but the plaid-patterning or non-contiguity is retained (Figure 2B, middle and right column).

Crucially, beyond delineating salient interaction patterns, we noticed how specific enclaves along the BPT highlighted topological features found in all BPTs. Typically, we observed that BPTs accumulated split branching at the top and then tended to have long streaks of strip branching toward their deeper end (Supplementary Figure S5). We reasoned we could use these ‘universal’ topological features to systematize our description of BPTs and thus directly address their topological diversity showcased earlier (Figure 1C). Briefly, we identified top enclaves as the deepest enclaves above which we only find split branching events; bottom enclaves as the highest enclaves below which we only find strip branching events and leaves as enclaves marking the end of the BPT (see Material and Methods). We verified the presence of these topological invariants with BPTs derived from Hi-C data with different resolutions (5 kb–1 Mb), from different cell types (IMR90, HUVEC and K562) and species (mouse and fly)(Supplementary Figure S5).

**Figure 3.**
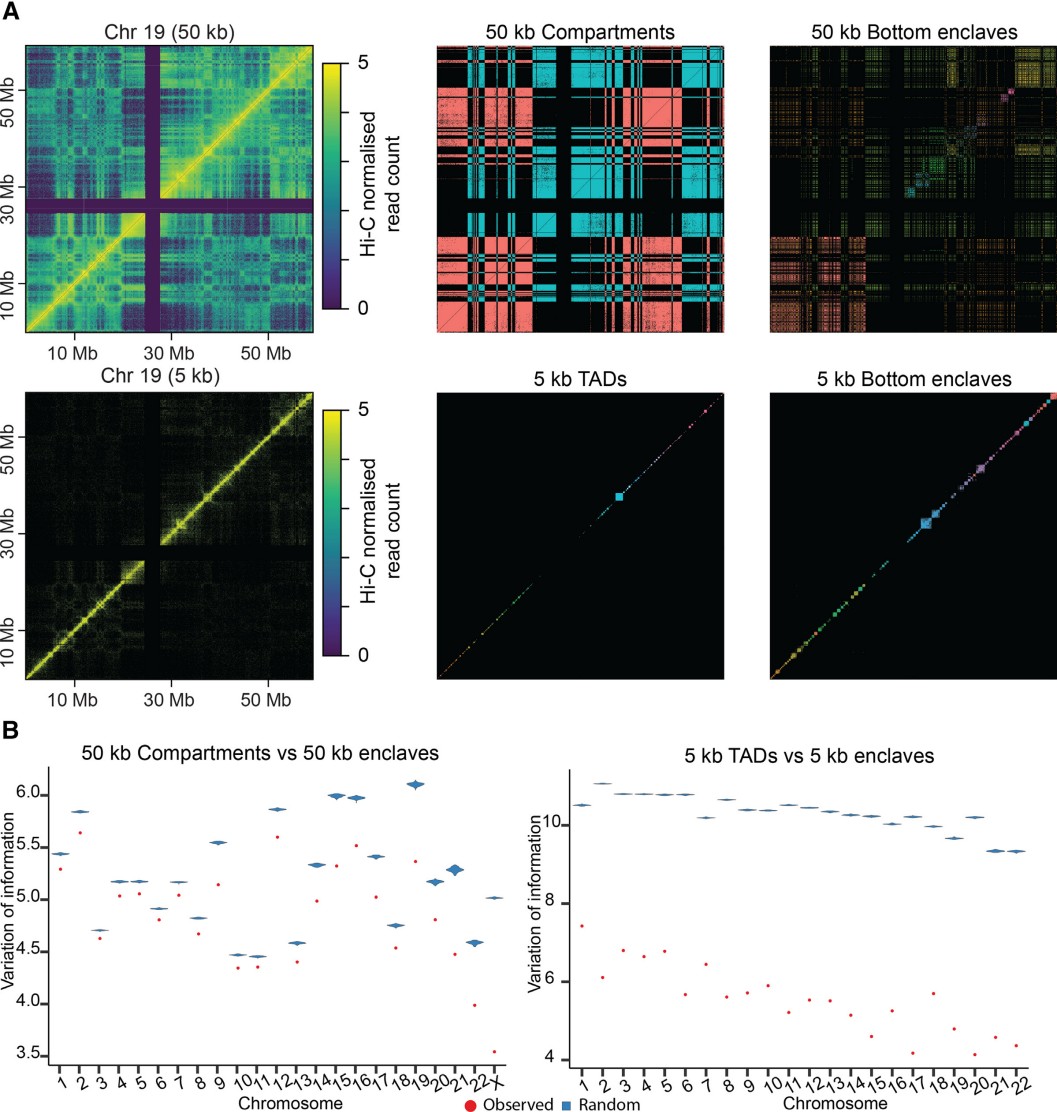
Enclaves agree well with both compartments and TADs. (**A**) Loci interaction heatmaps for 50 kb compartments (top middle) and 50 kb bottom enclaves (top right), 5 kb TADs (bottom middle) and 5 kb bottom enclaves (bottom right). Different colors are assigned to different clusters. Bottom enclaves coincide with different structures depending on the input data, showcasing how BHi-Cect dynamically adjusts the scale of interaction patterns it delineates based on the salient chromosome structure present in the input data. For local structures like TADs BHi-Cect further allows to characterize their inner arrangement, which highlights a variety of interaction patterns (Supplementary Figure S6). (**B**) Variation of information distance between enclaves and compartments (top, red), and between enclaves and TADs (bottom, red) in each chromosome. For comparison, distances between 500 randomly generated loci clusters and compartments (top, blue) or TADs (bottom, blue) are also shown. The smaller distance observed for enclaves (red) compared to random clustering (blue) at each chromosome suggests a significant agreement between our enclaves and known clusters (compartments or TADs).

Next, we examined the agreement of enclaves with compartments (16) and TADs (4) by computing the well-established variation of information metric (VI) (27) between our enclave clustering and these benchmark clustering. Briefly, VI evaluates the level of agreement between two clusterings based on information theoretic quantities (see Materials and Methods). Our VI analysis confirmed that this agreement was significant (*P*-value < 0.01) (Figure 3B) and supported the visual agreement observed between bottom enclaves and TADs or compartments when comparing their respective heatmaps (Figure 3A). The same trend was observed when using the average overlap metric (Supplementary Figure S3). We further noticed that BHi-Cect could characterize the inner arrangement of TAD-like clusters into further nested clusters with a variety interaction patterns including, but not restricted to sub-TAD like structures (Supplementary Figure S6). We also observed that this agreement extended to DNA loops. We evaluated the integration of DNA loops by computing the proportion of anchor sites pairs, characterizing a given DNA loop, that remained together within the same bottom enclave (see Materials and Methods). We noticed that with this metric, bottom enclaves by containing 79.9% of DNA loop anchor-site pairs, integrated DNA loops better than TADs (59.9%) detected at the same resolution (5 kb). Put together these observations support the notion that our enclave-based clustering integrates well with the current description of chromosome structure based on compartments, TADs or DNA loops.

### BHi-Cect segments the chromosome into functionally different clusters

**Figure 4.**
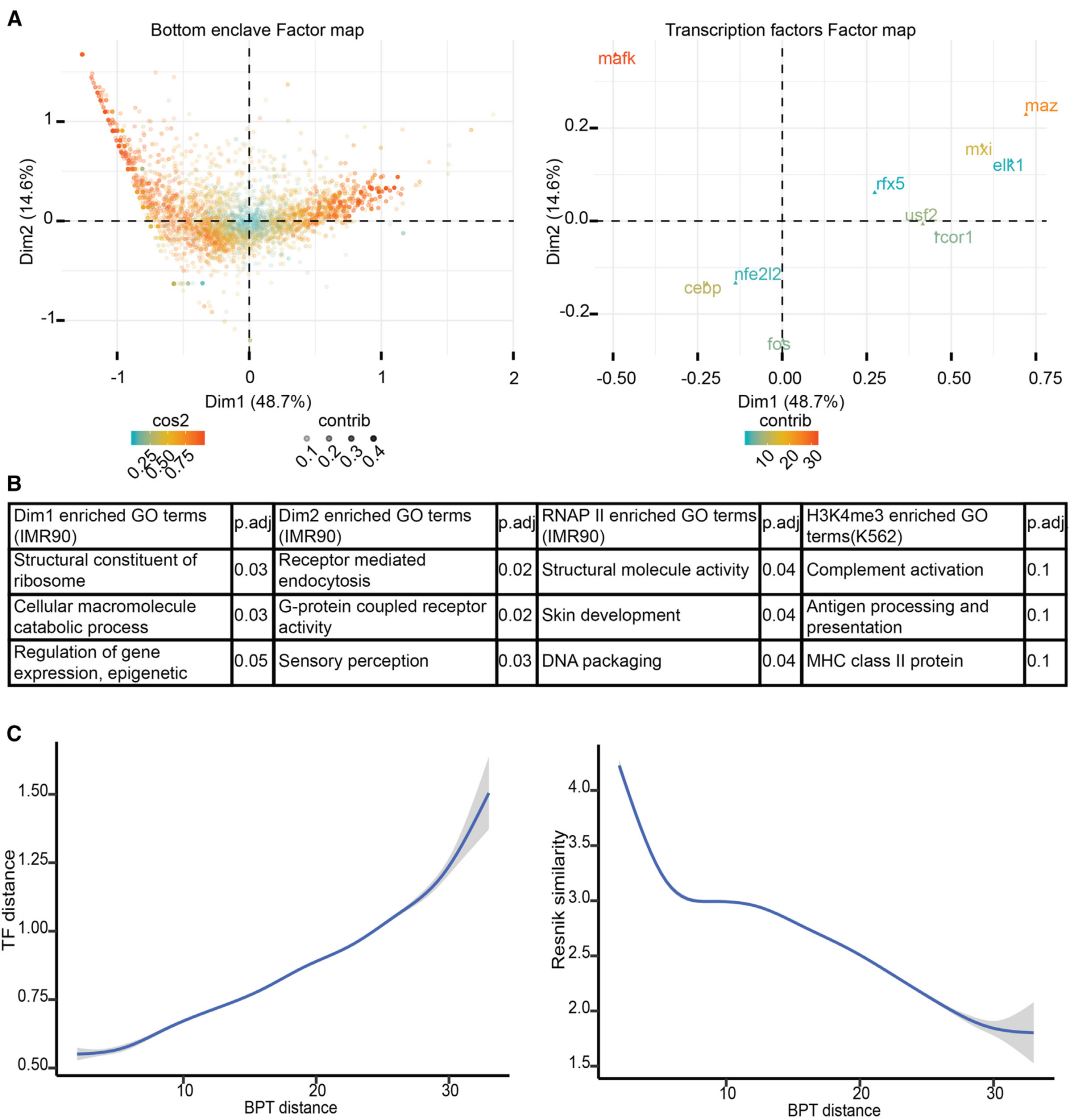
Bottom enclaves represent distinct functional units in terms of transcription factor and gene content. (**A**) Genome-wide factor maps derived from the correspondence analysis (CA) performed on the contingency table summarizing the transcription factor content in terms of number of transcription factor binding sites found within each bottom enclaves detected by BHi-Cect in the IMR90 human Hi-C data at 5 kb resolution. For this analysis we considered the binding sites for CEBP, RCOR1, MAZ, MXI, RFX5, MafK, FOS, USF2, NFE2L2 and ELK1. We can notice how bottom enclaves display a spectrum of transcription factor composition, indicating a variety of regulatory contexts. (**B**) Representative GO-terms enriched by bottom enclaves according to the epigenomic context they form. This enrichment was derived from a gene set enrichment analysis (GSEA) where we rank genes based on the epigenomic feature value of their respective bottom enclaves. Here, we present the enrichment observed with respect to the bottom enclave's position along the first and second dimension derived from the TF-content based CA previously described (first two tables); the enrichment with respect to the bottom enclave's number of RNA polymerase II binding sites (3rd table); the last table illustrates the enrichment observed when performing this same analysis with respect to the H3K4me3 content of bottom enclaves found in leukemic cell samples (K562) at 5 kb resolution. We report for all enrichments the corresponding FDR adjusted *P*-value (*P*.adj).(**C**) Trend lines summarizing the relation between functional similarity and structural proximity for bottom enclave pairs. Structural proximity (x-axis) corresponds to the minimum number of branches needed to link the considered pair of bottom enclaves in their associated BHi-Cect tree. Functional similarity on the left plot corresponds to the euclidean distance between the considered bottom enclaves in the reduced space formed by the first two dimensions obtained from the TF-content based CA previously described. Functional similarity on the right plot corresponds to the semantic similarity (Resnik similarity) in terms of the significantly enriched GO-terms found in each of the considered bottom enclaves respectively.

Following up on the observation that bottom enclaves coincided well with benchmark clusters (Figure 3A), we explored their potential biological relevance. We first examined the relation between the epigenomic context and the gene content of bottom enclaves. For this analysis we used the BHi-Cect results obtained at 5 kb resolution in IMR90 samples, to minimize the effect of binning. We summarized the epigenomic context of a given bottom enclave by computing the number of overlapping binding sites for a representative set of 10 transcription factors (TF) (see Material and Methods). We focused on characterizing the bottom enclave's TF content, because it outlines the regulatory context controlling the corresponding bottom enclave's gene content. We next characterized the variety of regulatory contexts formed by the different bottom enclaves as captured by their respective TF content. For this, we performed a correspondence analysis (CA) on the contingency table summarizing the number of binding sites for every TF found in each bottom enclave. Correspondence analysis is an extension of principal component analysis typically used to explore relationships between categorical variables, here bottom enclave and TF (see Materials and Methods). We found that the variety of TF contents found across bottom enclaves could be well summarized by the first two CA dimensions, accounting for 63.3% of the total variance (Figure 4A). Critically we noticed that bottom enclaves’ TF content could be described with respect to their relative position along two main ‘gradients’ in the reduced space formed by the first two CA dimensions (Figure 4A, left). Each of these gradient is characterized by a distinct set of TFs (MAFK vs. MAZ-MXI-RCOR1 and CEBP-FOS versus MAFK) and thus indicates that bottom enclaves vary mostly according to the relative prevalence in these distinct TFs (Figure 4A, right).

We next evaluated the extent to which the TF content of bottom enclaves associated with particular GO-terms through the GO annotation of the corresponding bottom enclaves’ genes. More specifically we performed a gene set enrichment analysis (GSEA) where we ranked genes with respect to their corresponding bottom enclave's position along either the first or second CA-dimension (see Materials and Methods). We found that bottom enclaves characterized by an increased prevalence of MAZ, MXI and RCOR1, associated with GO-terms broadly corresponding to housekeeping functions (Figure 4B, first table). In contrast bottom enclaves characterized by an increased prevalence of MAFK, associated with GO-terms broadly corresponding to environmental or immune response (Figure 4B, second table). This highlights how bottom enclaves coincide with very distinct functions, the more comprehensive tables reporting these enriched GO-term sets can be seen in Supplementary Tables S3 and S4. For comparison, when comparing these GSEA results with results obtained by ranking genes based on their expression level, we couldn’t retrieve as significant results and, as expected, mostly found enrichment in ribosome functions (Supplementary Table S2).

We extended this analysis by next characterizing the GO-term enrichment associated with the prevalence of RNA polymerase II (RNAP2) in bottom enclaves. The GSEA results based on bottom enclave RNAP2 prevalence highlighted skin function, structural molecule activity (cytoskeleton) and DNA packaging (Figure 4B, third table). We found these functions to be consistent with the fibroblastic nature of the IMR90 cell line used for this analysis. We further found that applying this analysis based on H3K4me3 (active histone mark) prevalence in bottom enclaves derived from K562 Hi-C data (5 kb) highlighted mostly immune functions, which conforms well with the leukemic state of K562 cells (Figure 4B, fourth table). The more comprehensive tables reporting these enriched GO-term sets can be seen in Supplementary Tables S5–S6. We validated these results by running a similar GSEA using the same epigenomic marks (RNAP2 and H3K4me3) in the same samples (IMR90 and K562 respectively) but this time ranking genes based on the prevalence of these marks in the corresponding promoter regions. The results of these GSEA were more numerous in terms of significantly enriched GO-terms but also more heterogeneous (Supplementary Tables S7 and S8).

We then examined whether this structure-function coupling could be extended beyond individual bottom enclaves by considering the BPT linking them. More specifically we evaluated how the BPT distance between pairs of bottom enclaves related to their corresponding functional similarity. Firstly we measured BPT distance by computing the minimum number of BPT branches required to join the considered bottom enclave pair. Secondly, we computed functional similarity based on the bottom enclaves’ TF content. Briefly, we computed the first two CA-dimensions derived from the contingency table summarizing the bottom enclave TF-content, as described earlier. We then computed the euclidean distance between the considered bottom enclave pair in that reduced space (TF-distance), to measure their functional similarity. When plotting BPT distance against the corresponding TF-distance we found a clear positive relation (Figure 4C, left). We validated this relation by swapping the BPT distance for the chromosome coordinate distance between the same pairs of bottom enclaves (see Materials and Methods) and found the relation to be weaker as quantified by a Pearson correlation test (Supplementary Table S1). We further confirmed this relation by swapping the TF-distance between bottom enclaves with the semantic similarity (Resnik similarity) in terms of the enriched GO-terms found respectively in each of the members of the considered bottom enclave pair (see Materials and Methods). Remarkably, 72% of bottom enclaves (3128/4361) displayed significantly enriched GO-terms and we also found the same relation, with the semantic similarity decreasing as we consider bottom enclaves located further apart in the BPT (Figure 4C, right). These results indicate that the overall chromosome architecture captured by the BPT also is coupled with the functionality of the chromosome regions it clusters.

### The position along the BPT coincides with DNA activity

To extend our analysis beyond bottom enclaves, we next examined the biological relevance of the BPT as a whole. More specifically, we analyzed the relation of the chromosome's hierarchical structure, as captured by the whole BPT, with the biology of the underlying DNA. To this end, we systematically explored how different epigenomic features varied as we considered loci present at different positions or depths along the whole BPT. We quantified this depth based on the branching events that shape our BPT. Briefly, we defined a score, which we call ‘nestedness’, for each locus accounting for the number of branching events necessary to reach the locus relative to the total number of branching found in the considered BPT (see Materials and Methods). The reason we talk of nestedness, relates to the observation that branching events indicate the presence of some loci further preferentially self-interacting within the already found cluster. This suggests that the degree of confinement or nestedness for every locus within the chromosome architecture can be evaluated from the number of associated branching events. We further needed our nestedness score to universally apply to every BPT despite their characteristic topological diversity (Figure 1C). To achieve this, we will leverage the topological invariants identified earlier (top/bottom enclaves and BPT leaves). Briefly, we set the nestedness score of these BPT positions to 0 for top enclaves, 0.5 for bottom enclaves and 1 for BPT leaves. We then linearly interpolate nestedness scores for any intermediary BPT positions (see Materials and Methods).

**Figure 5.**
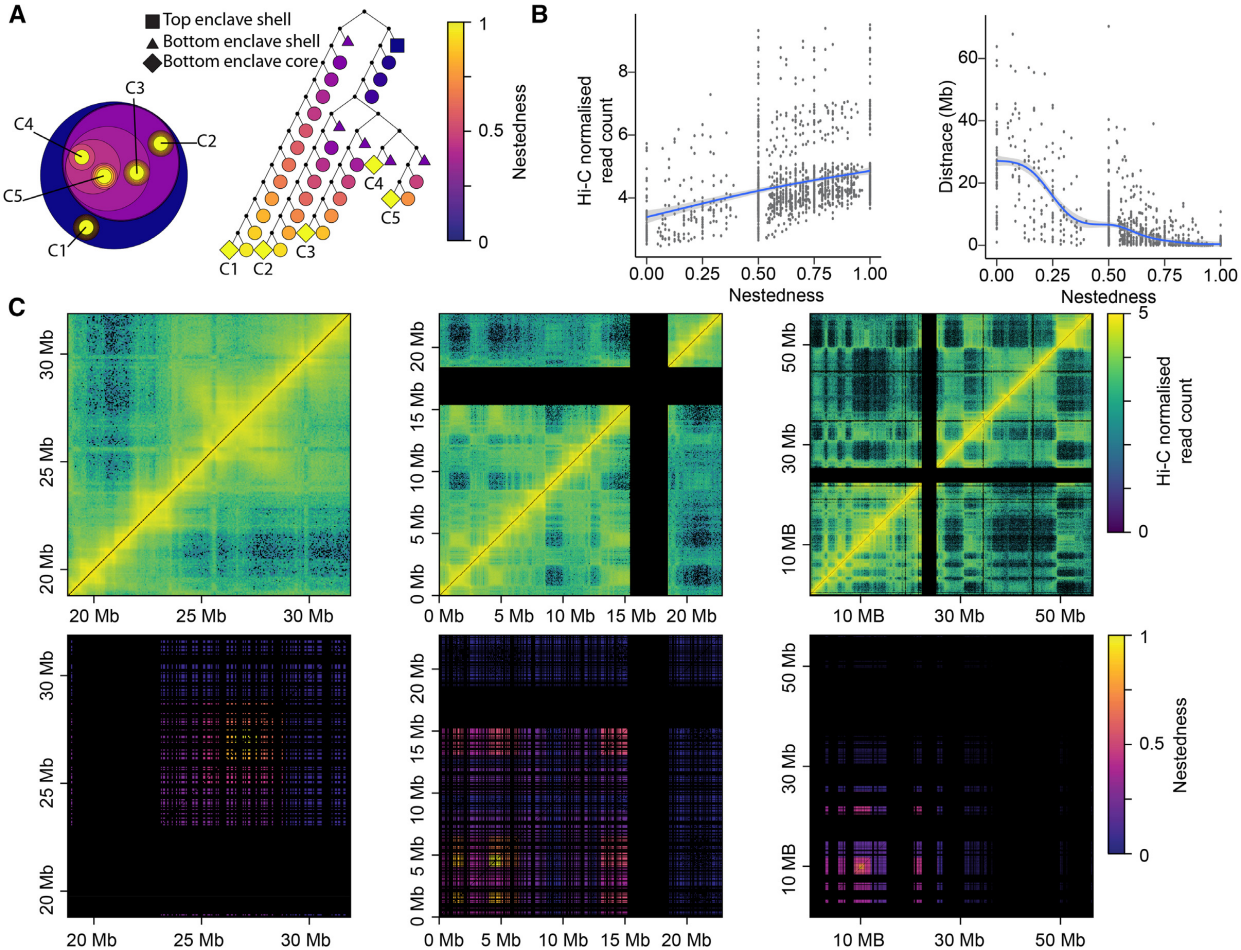
Nestedness as a universal measure of a locus’ position along their respective BHi-Cect partition tree. (**A**) Illustration of the nestedness metric with representations of the BPT as nested clusters (left) and a tree (right). The color reflects the clusters’ nestedness values defined for every locus within it to reflect their relative position with respect to top and bottom enclaves. The different tree node shapes highlight the important landmarks in the BPT hierarchical structure. The clusters (left) and nodes (right) labeled C1-C5 indicate the bottom enclave cores. (**B**) Trend line for the average interaction frequency (left) and the genomic span (right) found in BPT clusters along their nestedness. The trend line is the result of a locally weighted scatterplot smoothing (LOESS) fit and the grey region indicates its 95% confidence interval. (**C**) Examples of bottom enclave heatmaps highlighting how nestedness is distributed along genome coordinates from chromosome 18 (first two enclaves) and 17 (right-most enclave) from IMR90 Hi-C data (50 kb). We can notice how enclaves display a variety of span and contiguity across all levels of nestedness.

Using this score on 50 kb IMR90 Hi-C data, we observed that the highly nested regions in enclaves tended to be accumulated in relatively narrow genomic regions spanning hundreds of kb with relatively higher interaction frequencies (Figure 5B and C). In contrast, less nested regions spanned tens of Mb with relatively lower interaction frequencies.

**Figure 6.**
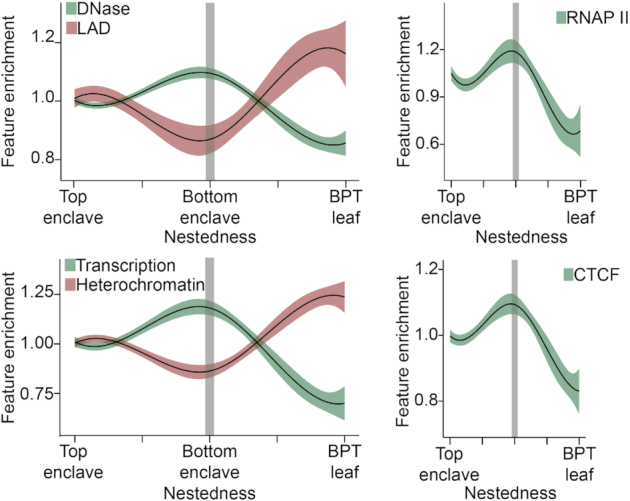
Loci nestedness is coupled with epigenomic activity We plot here the epigenomic feature enrichment for each locus as a function of their BPT nestedness. The trends for DNase accessibility against lamina associated domain (LAD) enrichment (top-left) and transcription against heterochromatin histone marks based on chromHMM labels enrichment (bottom-left)are shown. The trends for RNAP II and CTCF enrichment are shown independently (right column). The trend lines were obtained using b-spline smoothing with 4 degrees of freedom and the shaded areas mark the corresponding 95% confidence intervals.

Next, we explored the physiological implications of nestedness by comparing it with previously reported epigenomic datasets (Figure 6). To comprehensively capture the multiple facets of epigenomic activity we considered the following epigenomic features: DNase accessibility, lamina associated domain (LAD), histone modifications as integrated by chromHMM annotations (34), CAGE clusters, short and long read RNA-seq peaks, DNA-binding factor binding site for 10 representative TFs, RNAP2 and three chromatin remodeling factor binding sites (CTCF, RAD21 and CHD1).

When examining the epigenomic influence of nestedness across the whole BPT we noticed a clear antagonism between top and bottom enclave trends (Figure 6). Within top enclaves, we observed a progressive enrichment in epigenomic activity with nestedness. This is indicated by increased enrichment in active DNA marks such as DNase accessibility or transcription chromHMM annotations matched by a depletion of inactive DNA marks, such as LAD overlap or heterochromatin chromHMM labels, as we consider increasingly nested loci. This top enclave trend contrasts with the progressive depletion in epigenomic activity with increased nestedness observed within bottom enclaves. This antagonism results in loci present at the boundary between top and bottom enclaves, in terms of nestedness, concentrating the relatively more active regions within the chromosome. Furthermore we noticed that the long range interactions harbored by boundary-loci coincided with a significant enrichment in chromatin remodeling factors like CTCF (Figure 6 bottom right), indicating that active looping mechanisms are bringing those distant genomic regions together. These trends suggest that the boundary loci correspond to the active and open chromatin regions of the chromosome. These trends were further observed in human umbilical vein endothelial and K562 (leukemic) cell lines suggesting their generalizability (Supplementary Figure S10). We further validated these trends by running the same analysis with randomly generated BPT clustering of the corresponding Hi-C data and observed a complete loss of the trends described above (Supplementary Figure S11).

We also noticed that the average number of overlaps between the considered epigenomic features and the loci of interest provided the most consistent trend along nestedness (Supplementary Figure S7) compared to average ‘intensity’ of the epigenomic features, whether it be average ChIP-Seq, CAGE or RNA-seq peak intensity (Supplementary Figure S8) or overlap size for epigenomic annotation (Supplementary Figure S9). This indicates that nestedness is most correlated with the density of epigenomic activity found across chromosome loci. The significance of the observed enrichment or depletion is reported in Supplementary Table S9.

## DISCUSSION

This paper introduces BHi-Cect, a spectral clustering method to chart insulated, non-contiguous and nested chromosome clusters, which we map using our novel BPT framework. We developed a hierarchical description of chromosome structure, that provides key insights into chromatin architecture.

Previous methods using spectral clustering applied to Hi-C focused on determining the scale at which the found clusters best matched already known clusters such as TADs or compartments (14,15,17,35–37). Our clustering method augments existing structural descriptions by using the BPT representation for chromosome structure, which efficiently captures the diversity of hierarchical relations bridging the variety of preferentially self-interacting clusters that make up the chromosome.

A unique feature of BHi-Cect is how it dynamically delineates chromosome clusters that emphasize the salient interaction patterns found in the input data. Notably, we show that with low resolution Hi-C data (50 kb), BHi-Cect highlights global clusters spanning the whole chromosome while with high resolution Hi-C data (5 kb), BHi-Cect will focus on local chromosome clusters spanning less than 1 Mb. We show how these different clustering outcomes are self consistent and agree well with corresponding global (compartments) and local (TADs) benchmark chromosome clusters.

A key finding are enclaves, which are a novel form of chromosome clusters identified by our method, that are mostly non-contiguous and deeply interwoven. Critically our method targets preferentially self-interacting clusters expected to be arranged as a tightly packed ‘core’ loci-cluster nested within a looser ‘shell’ of long-ranging DNA loops. Enclaves complement TAD-based description of chromosome structure by offering a much more diverse and flexible range of possible conformations that supports the irregular chromosome organisation suggested by others, notably in a recent electron microscopy study (38). In this framework the overall structure of chromatin is shaped by different levels of compaction that generate 3D nuclear domains in which DNA is made more or less concentrated and thereby accessible by looping the flexible DNA fiber at various lengths and creating contacts between and within distant chromosome regions.

Bottom enclaves are clusters found in all BPTs and capture key aspects of the structure-function coupling at play in the genome. Our CA results highlight how the bottom enclave's TF-content shapes distinct regulatory regimes. We found that the prevalence of MAZ, MXI, USF2 and RCOR1 in bottom enclaves indicated a ‘steady’ regulatory regime typically associated with housekeeping genes. In contrast, we found that the prevalence of MAFK in bottom enclaves indicated a ‘dynamic’ regulatory regime typically associated with environmental or immune response genes. This illustrates how chromosome clusters can form quite distinct regulatory micro-environments for the genes contained within them. GSEA results focusing on the prevalence of RNAP2 and H3K4me3 in bottom enclaves, indicated the colocalization of genes whose function directly contributed to biological functions of the considered cell lines (cytoskeleton and skin function enrichment in IMR90 fibroblast samples or immune function enrichment in K562 leukemia samples). Since the prevalence of RNAP2 or active histone marks (H3K4me3) promote transcription, this trend could indicate a direct involvement of chromosome structure in the functional specialization of the cell, by setting distinct micro-environments for specific gene sets to activate particular biological processes. Furthermore we argue that the more focused and biologically relevant set of functions highlighted by the 3D regulatory context provided by bottom enclaves supplements the more significant but heterogeneous set of functions highlighted by the 1D regulatory context provided by promoters. Overall these results suggest an integral structure-function coupling between the regulatory context of bottom enclaves and the gene content of bottom enclaves.

Our analysis integrating loci nestedness and corresponding epigenomic features showed key implications stemming from our description of chromosome architecture. Inspired by early Hi-C results, revealing the correspondence between Hi-C data and a fractal globule conformation of the chromosome (8), we developed the notion of nestedness. Critically nestedness, or the notion that structural hierarchy in the chromosome is driven by the tendency of smaller clusters to be nested within larger clusters, significantly coincided with epigenomic activity (Figure 6). We show that along this hierarchy, the transition into bottom enclaves harbors the maximal density for active DNA marks and that the most nested clusters (BPT leaves) display the maximal density for inactive DNA marks. Coupled with the trends observed for LADs, DNase and chromatin remodeling factors (Figure 6), this could indicate that BPT leaves represent compact anchoring regions attached to the nuclear lamina, wrapped by a shell of DNA loops with chromatin remodeling factors (e.g. CTCF, RAD21, CHD1) co-localizing active chromosome regions into bottom enclaves to form transcription factory-like structures (39) (Supplementary Figure S13).

Our results also suggest that depending on the position along the enclave hierarchy the cluster is found, different mechanisms might be shaping the strong loci-interaction observed within it. Firstly, for both top and bottom levels of hierarchy, increased nestedness is driven by the average increase in interaction frequency between the considered loci (Figure 5B). Secondly, this trend draws different epigenomic implications depending on whether we consider top or bottom levels of chromosome hierarchy. At the top level, increase in interaction frequency enables the colocalization of biologically active DNA regions. This contrasts with the trend observed at the bottom level of hierarchy where an increase in interaction frequency along nestedness colocalizes loci enriched in inactive DNA marks and LADs. This antagonism between top and bottom enclaves in terms of what kind of DNA marks get brought together by increased interaction strength, implies that possibly different factors and mechanisms might be setting these strong interactions at these different hierarchical levels of chromosome structure. This notion was hypothesized and tested in a recent simulation study (40). In this paper they argue that loop extrusion is setting local structures while phase separation is the driver of global cluster formation in the chromosome. A similar dynamic equilibrium between competing molecular mechanisms could also explain the trends we observed, though the details of which cannot be inferred with the present analysis.

In conclusion, our method and the identified chromatin hierarchical structure may serve as a framework for drawing inferences from Hi-C and other advanced genome-wide studies toward unveiling key architectural principles guiding chromatin conformation. Further research should be done to examine how the arrangement of enclaves varies across tissues while integrating other known features of chromatin architecture, such as biophysical constraints derived from *in vitro* and molecular dynamics studies (41) or DNA binding factor content derived from molecular biology studies (42), to better inform the segmentation of the chromosome into more biologically realistic structures.

## DATA AVAILABILITY

The code for the algorithm is available at https://github.com/princeps091-binf/BHi-Cect.

## Supplementary Material

gkaa004_Supplemental_Files
