# Supplementary material for: BHi-Cect: a top-down algorithm for identifying the multi-scale hierarchical structure of chromosomes
Source: Nucleic Acids Res. 2020 Feb 3;48(5):e26. doi: 10.1093/nar/gkaa004 (PMC7049727; doi:10.1093/nar/gkaa004)
Supplement: gkaa004_Supplemental_Files [file gkaa004_supplemental_files.zip › Supplementary_figures.pdf]

Supplementary figuresloci

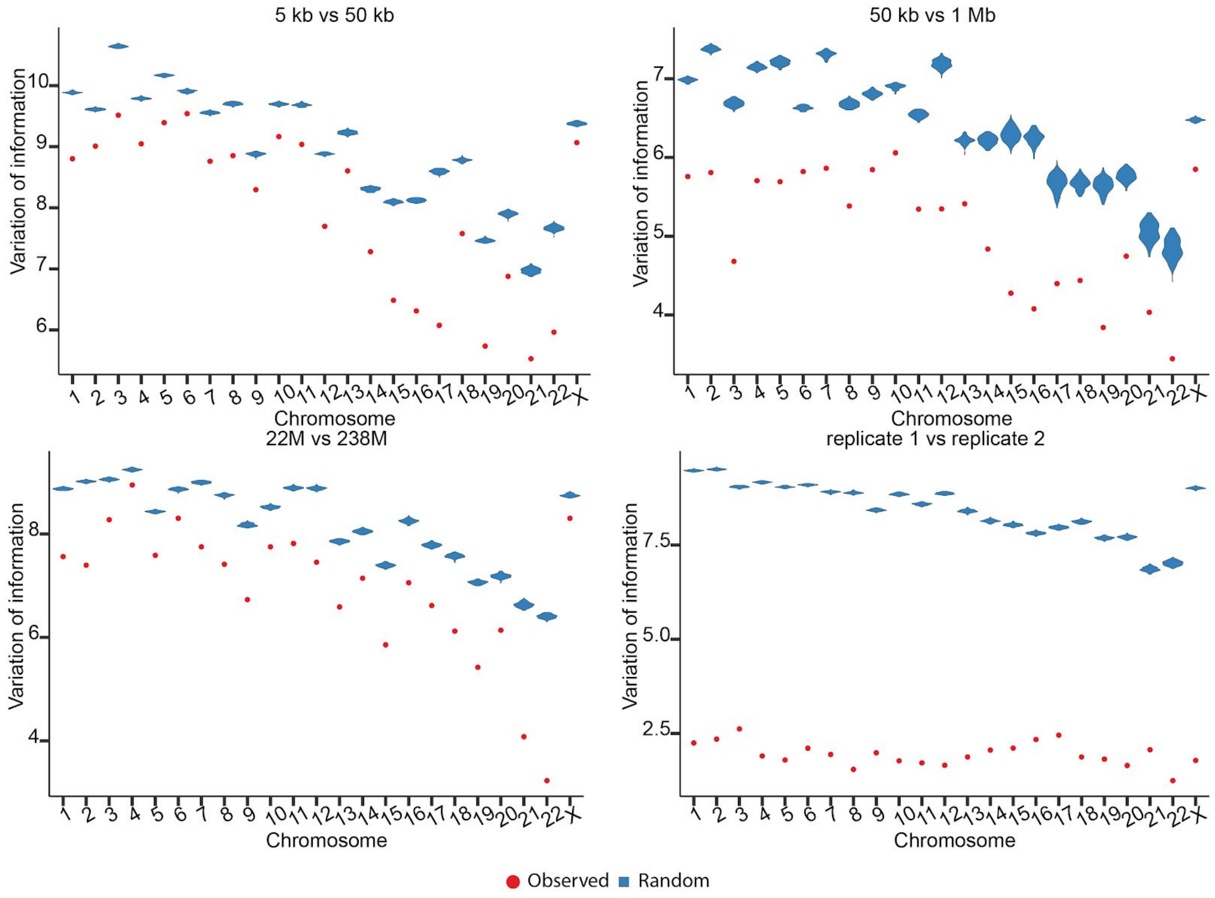

Supp. Figure 1: Variation of information distance between BHi-Cect clustering applied on IMR90 Hi-C data across different resolutions (1st row), and across different Hi-C read depths (bottom, left) and replicates (bottom right) in each chromosome. For comparison, distances with randomly generated loci clusters (blue violin plots) are also shown. The large difference between the observed and random clustering VI distances suggest a significant agreement between the compared datasets.

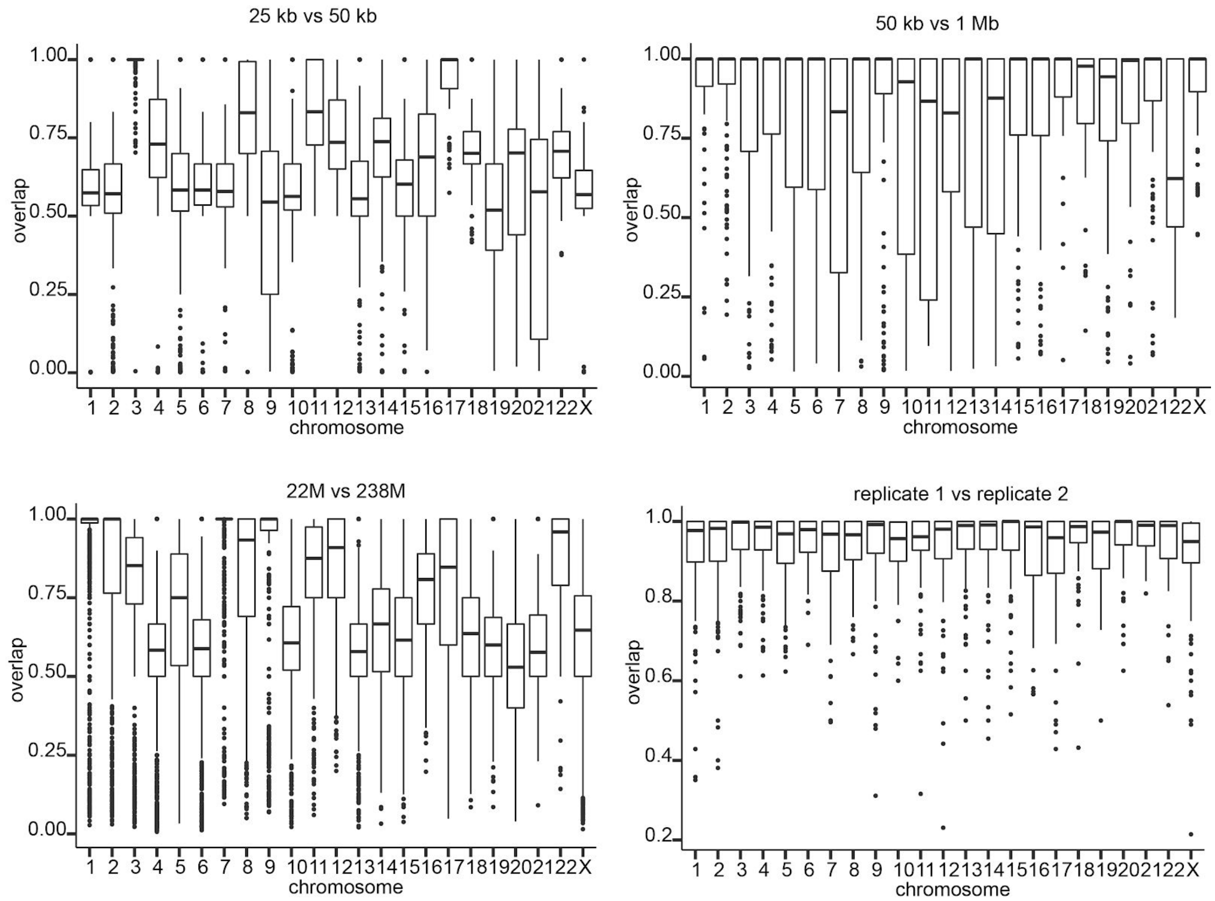

Supp. Figure 2: Percentage of overlap between BHi-Cect clustering applied on IMR90 Hi-C data across different resolutions (1st row), and across different Hi-C read depths (bottom, left) and replicates (bottom right) in each chromosome. The median overlap percentage being consistently above 50 % suggests a good level of agreement between the compared datasets.

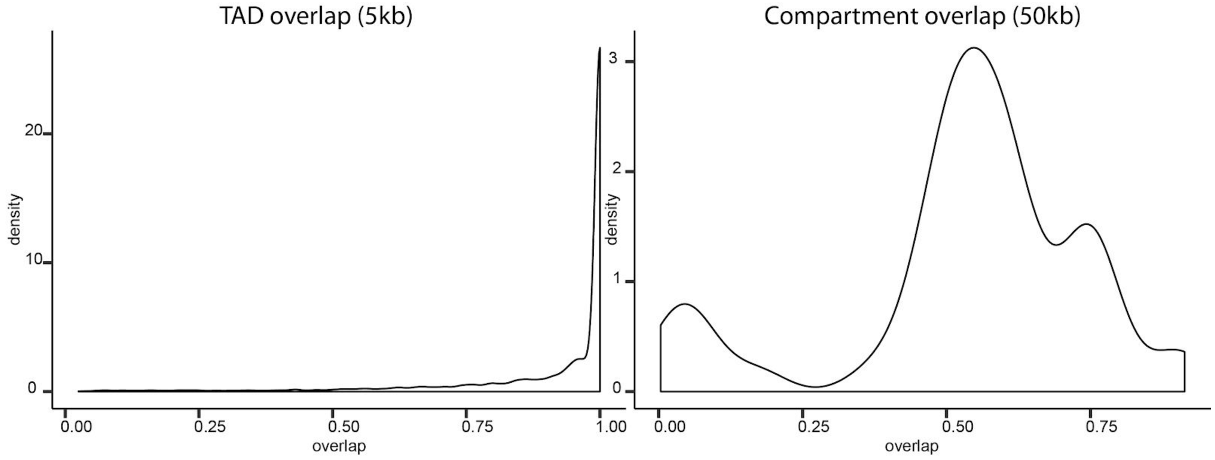

Supp. Figure 3: Density plots of the percentage of overlap between BHi-Cect clustering applied on IMR90 Hi-C data across different resolutions (5 kb and 50 kb) with TADs and compartments. The bulk

of the overlap percentage distribution being consistently above 0.5 suggests a good level of agreement between the compared clustering.

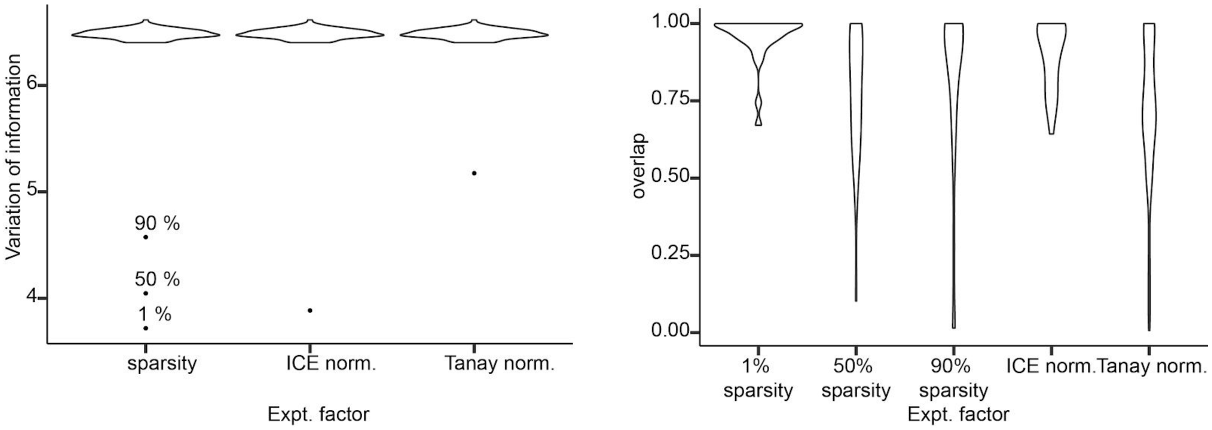

Supp. Figure 4: Robustness of BHi-Cect clustering towards Hi-C normalisation and sparsity. We artificially subset 1%, 50% and 90% of chromosome 22 IMR90 Hi-C KR normalised data at 50 kb resolution to emulate different sparsity levels. We then ran BHi-Cect onto these Hi-C datasets with different sparsity levels, ICE normalised data and Hi-C data normalised following the scheme described by Yaffe & Tanay. We then computed the variation of information distance between these different clustering and the original BHi-Cect clustering reported in the main manuscript (KR normalised IMR90 Hi-C data at 50 kb resolution). For comparison we also computed the variation of information distance between the original BHi-Cect clustering and random clustering. All conditions (sparsity and normalisation) observed consistently agree better than random clustering (violin plots), indicating the robustness of BHi-Cect towards these factors. ICE normalised clustering is more similar to KR normalised clustering than the Yaffe & Tanay normalised clustering. This illustrates how ICE and KR are both iterative proportional fitting algorithm expected to optimise for the same log linear model of Hi-C data. We also computed the percentage of overlap with the best matching clusters across these clustering and the original BHi-Cect clustering. We can see how these overlap percentages are consistent with the corresponding variation of information trends.

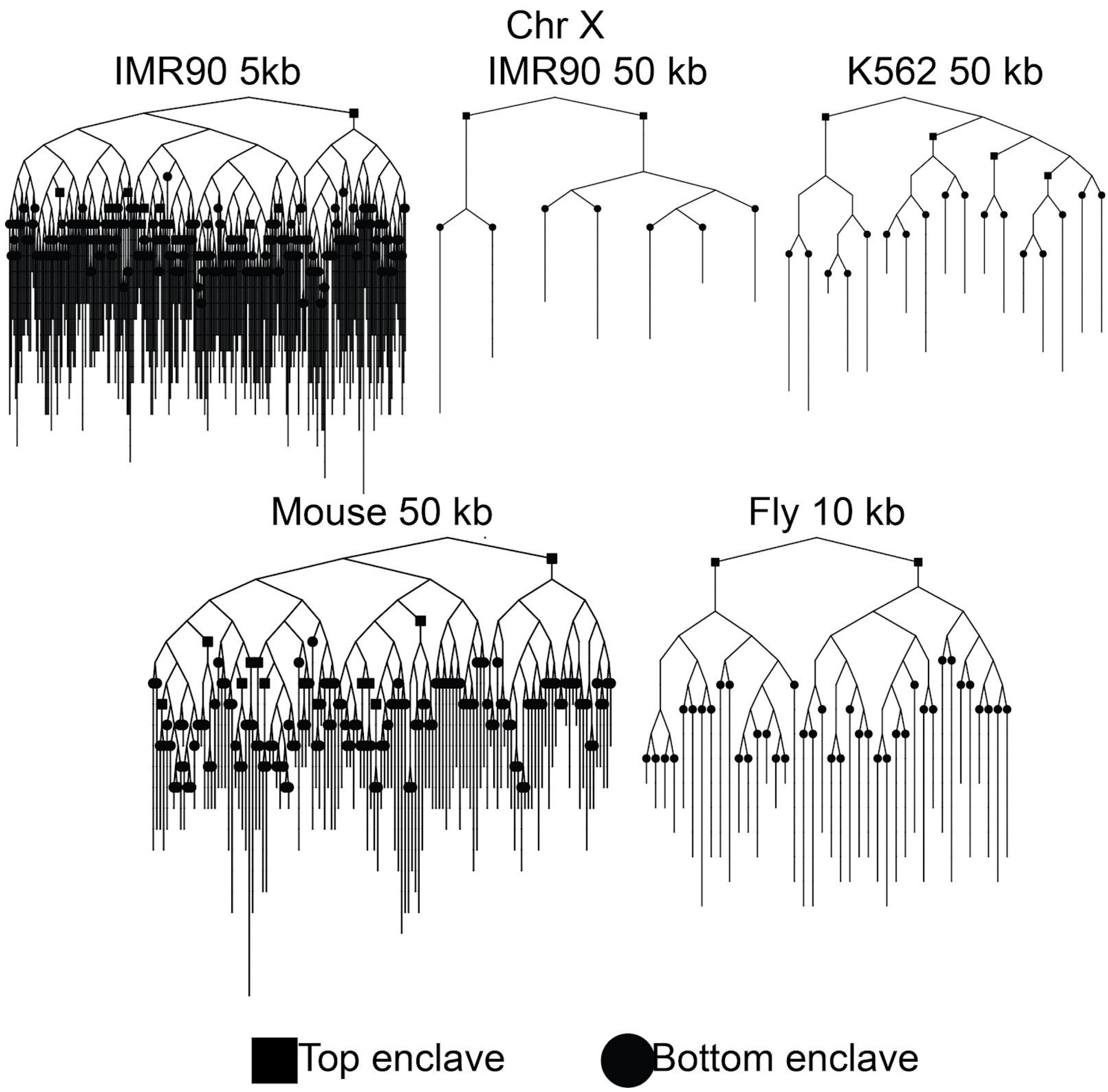

Supp. Figure 5: All BPTs harbour topological invariant features. BPTs obtained by running BHi-Cect on chromosome X across different resolutions, cell lines and species. For visibility we only plotted the BPT nodes corresponding to preferentially self-interacting clusters (expansion metric < 1). We can notice how all BPTs accumulate split branching events towards the top and strip branching events towards the bottom of the tree. We capture this topological feature by systematically reporting the tree nodes or enclaves marking the lowest positions above which we only find split branching events (top enclaves) and the highest positions below which we only find strip branching events (bottom enclaves).

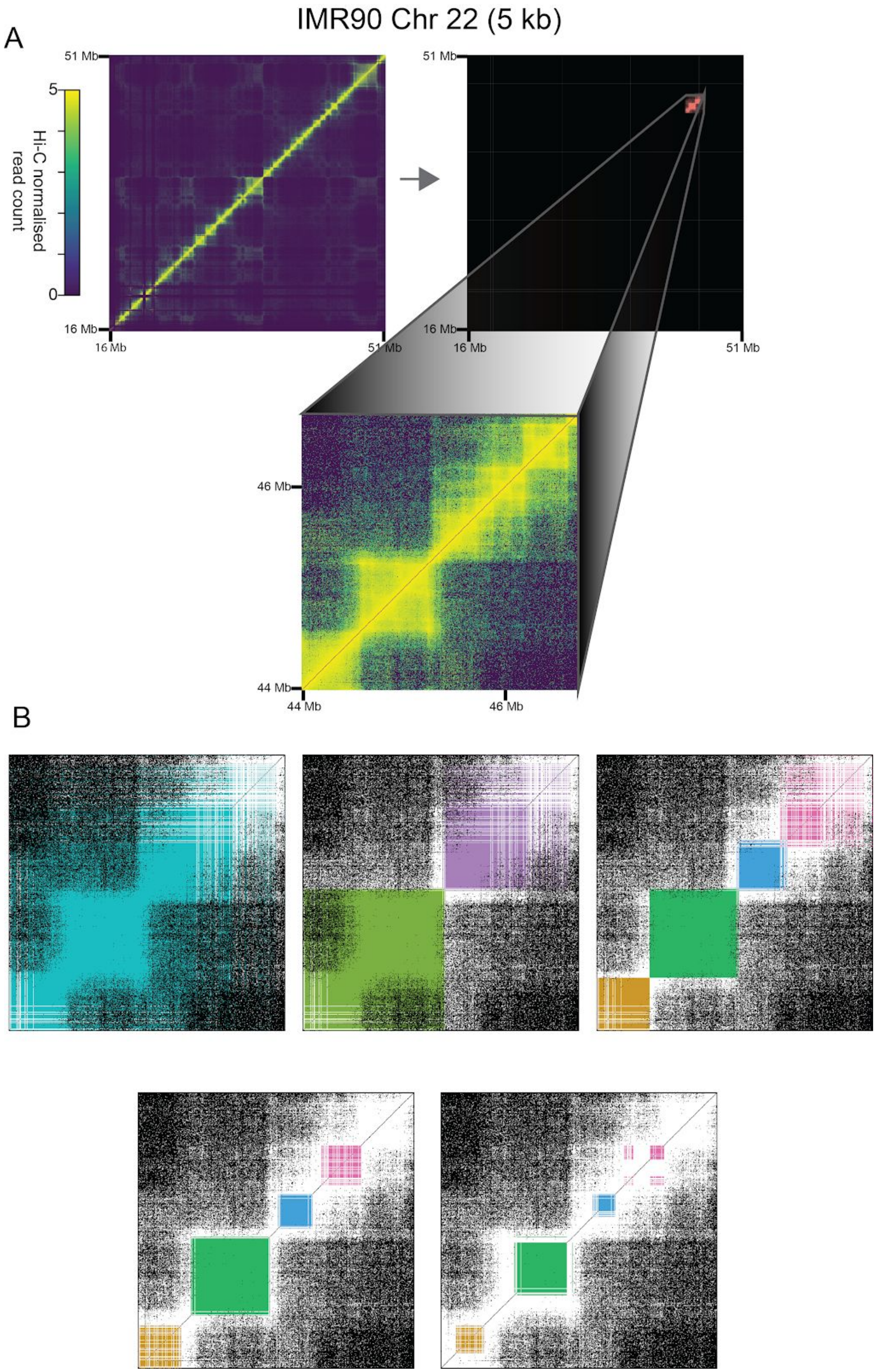

IMR90 Chr 22 (5 kb)
A
51 Mb
16 Mb
16 Mb
51 Mb
5
0
Hi-C normalised read count
51 Mb
16 Mb
16 Mb
51 Mb
46 Mb
44 Mb
44 Mb
46 Mb
B

Supp. Figure 6: BHi-Cect can also highlight sub-TAD like interaction patterns. A) Series of interaction heatmaps highlighting interaction patterns at different scales. The top row illustrates how BHi-Cect highlights salient interaction clusters when examining the whole chromosome. B) Further focusing on the cluster previously detected (A), we notice how BHi-Cect can highlight the inner interaction patterns shaping the delineated cluster. The white pixels indicate the interactions present in the original cluster but absent at the considered BPT position. Black pixels indicate no interactions. Colors were assigned so that different clusters (split branching events) are given unique colors, whereas, different levels of the same cluster (strip branching events) are given the same color as their closest parent cluster (split branching). Critically we find that BHi-Cect delineates both diagonal blocks (green clusters) as well as plaid interaction patterns (orange cluster). Furthermore BHi-Cect finds more complex patterns like off-diagonal streaks (pink cluster) that could indicate more intricate DNA looping interactions.

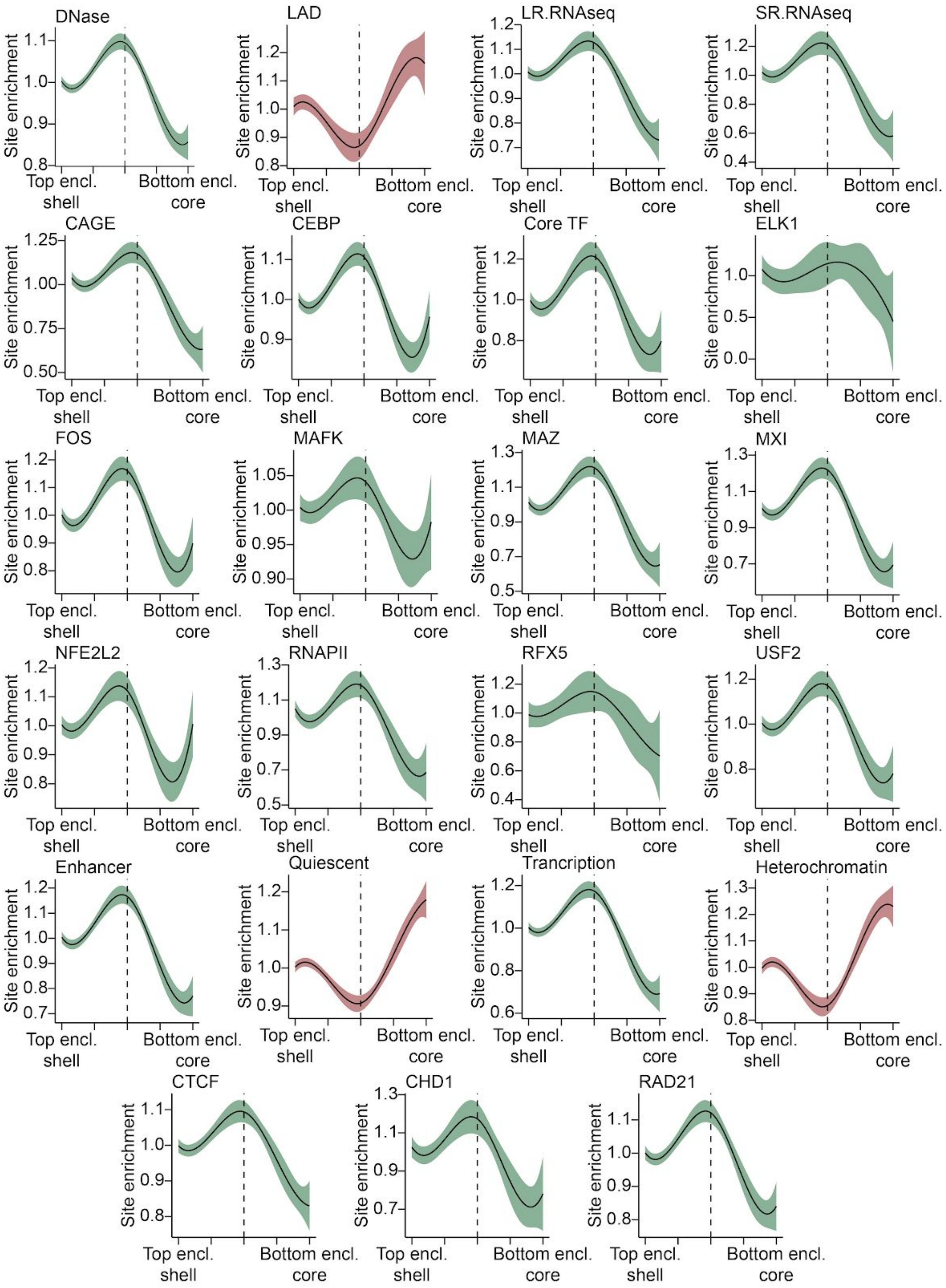
DNase
LAD
LR.RNAseq
SR.RNAseq
CAGE
CEBP
Core TF
ELK1
FOS
MAFK
MAZ
MXI
NFE2L2
RNAPII
RFX5
USF2
Enhancer
Quiescent
Trancription
Heterochromatin
CTCF
CHD1
RAD21
Site enrichment
Top encl. shell
Bottom encl. core

Supp. Figure 7: Epigenomic enrichment profile along BPT nestedness for all epigenomic features considered. Trend lines correspond to a spline fit with 4 degrees of freedom and the shaded region indicates their 95% confidence interval.

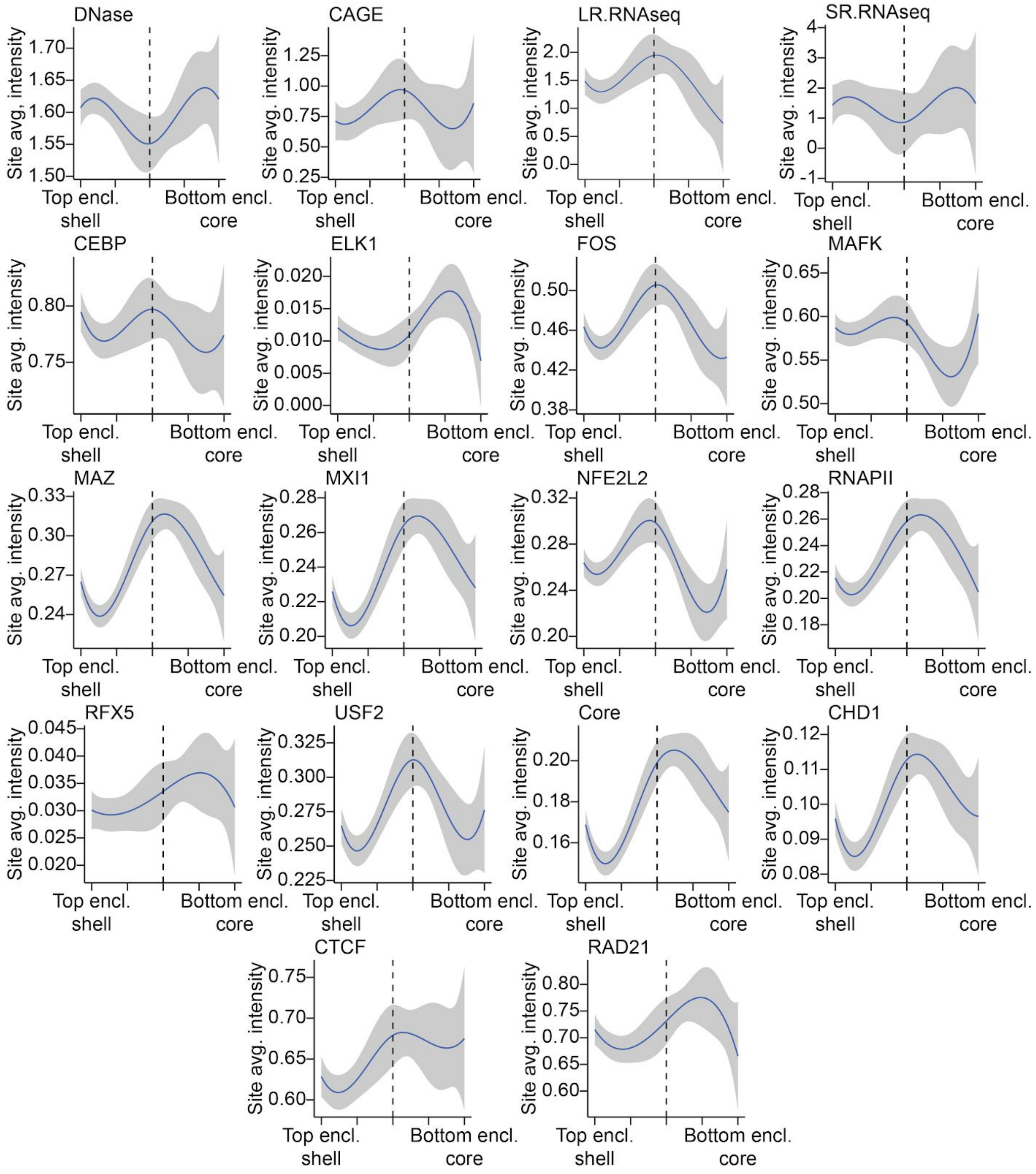

Supp. Figure 8: Epigenomic relative average intensity profile along BPT nestedness for all affinity-based epigenomic features considered. Trend lines correspond to a spline fit with 4 degrees of freedom and the shaded region indicates their 95% confidence interval.

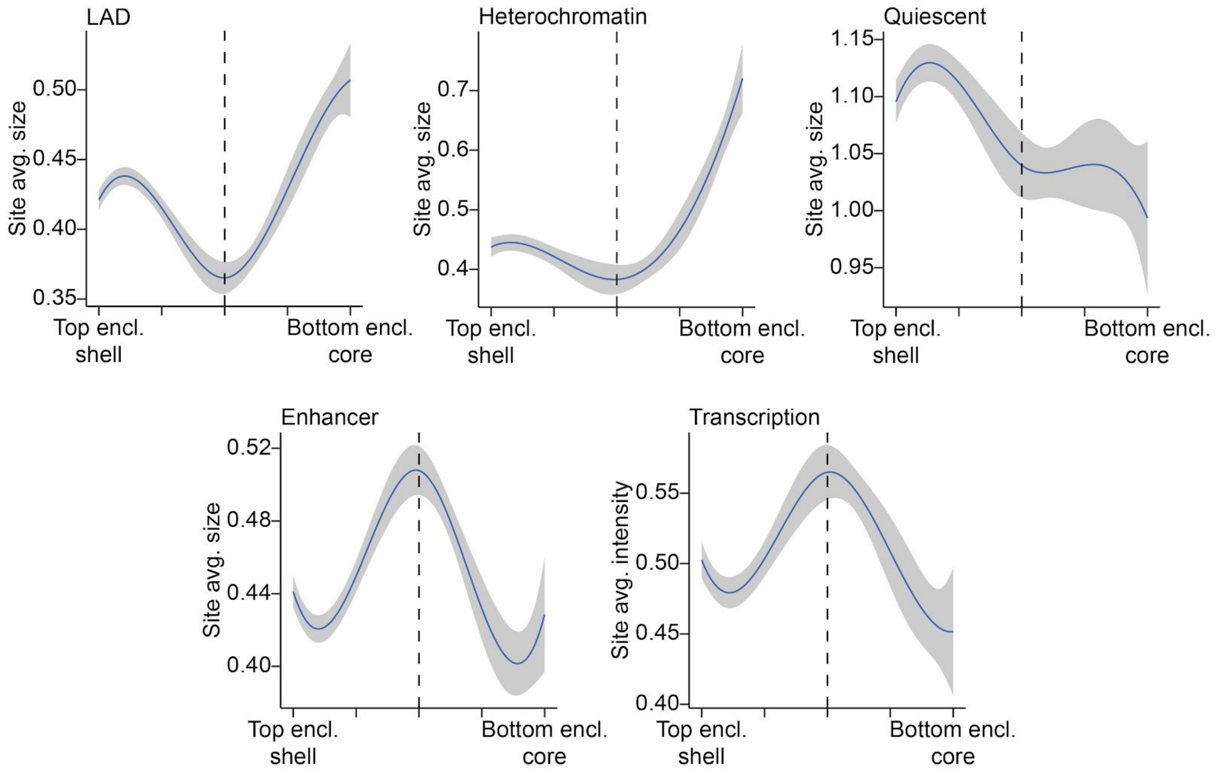

Supp. Figure 9: Epigenomic relative average feature size profile along BPT nestedness for all annotation epigenomic features considered. Trend lines correspond to a spline fit with 4 degrees of freedom and the shaded region indicates their 95% confidence interval.

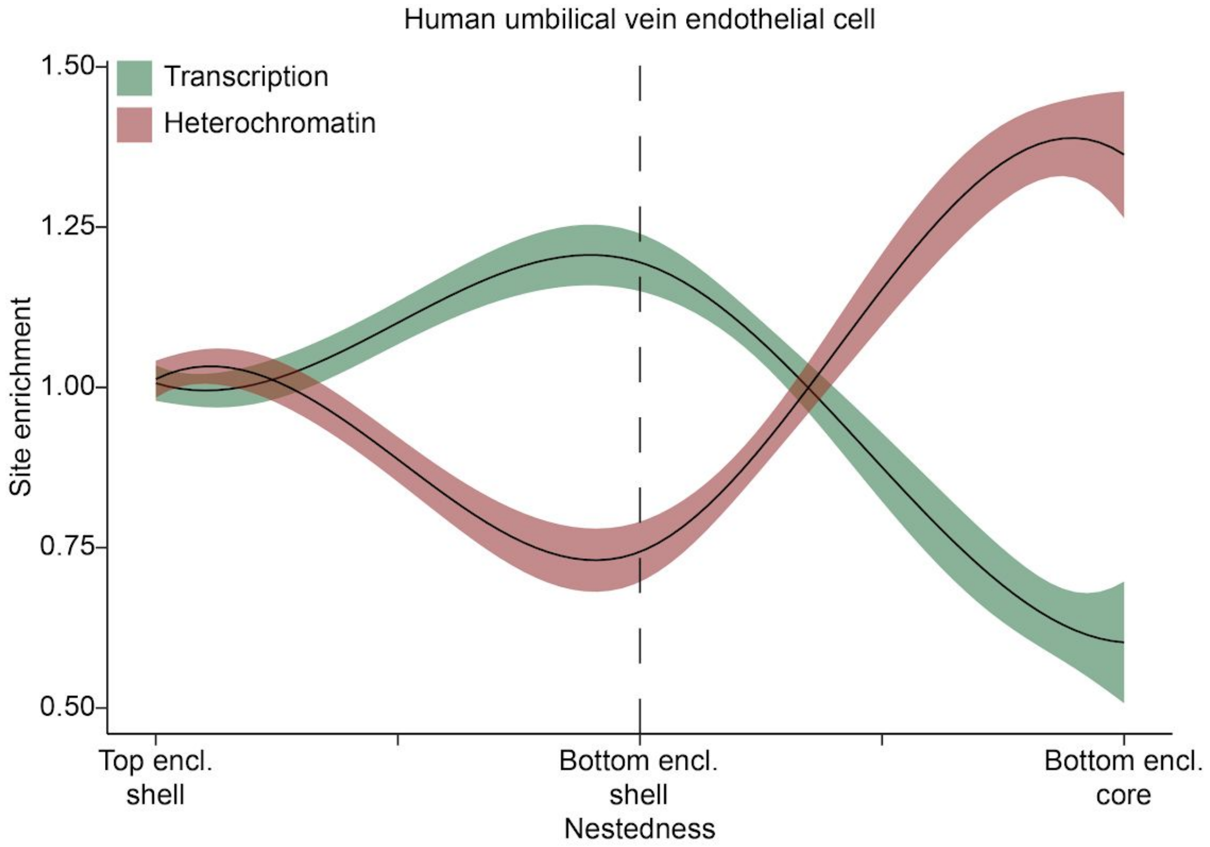
Human umbilical vein endothelial cell
Transcription
Heterochromatin
Site enrichment
1.50
1.25
1.00
0.75
0.50
Top encl. shell
Bottom encl. shell
Bottom encl. core
Nestedness

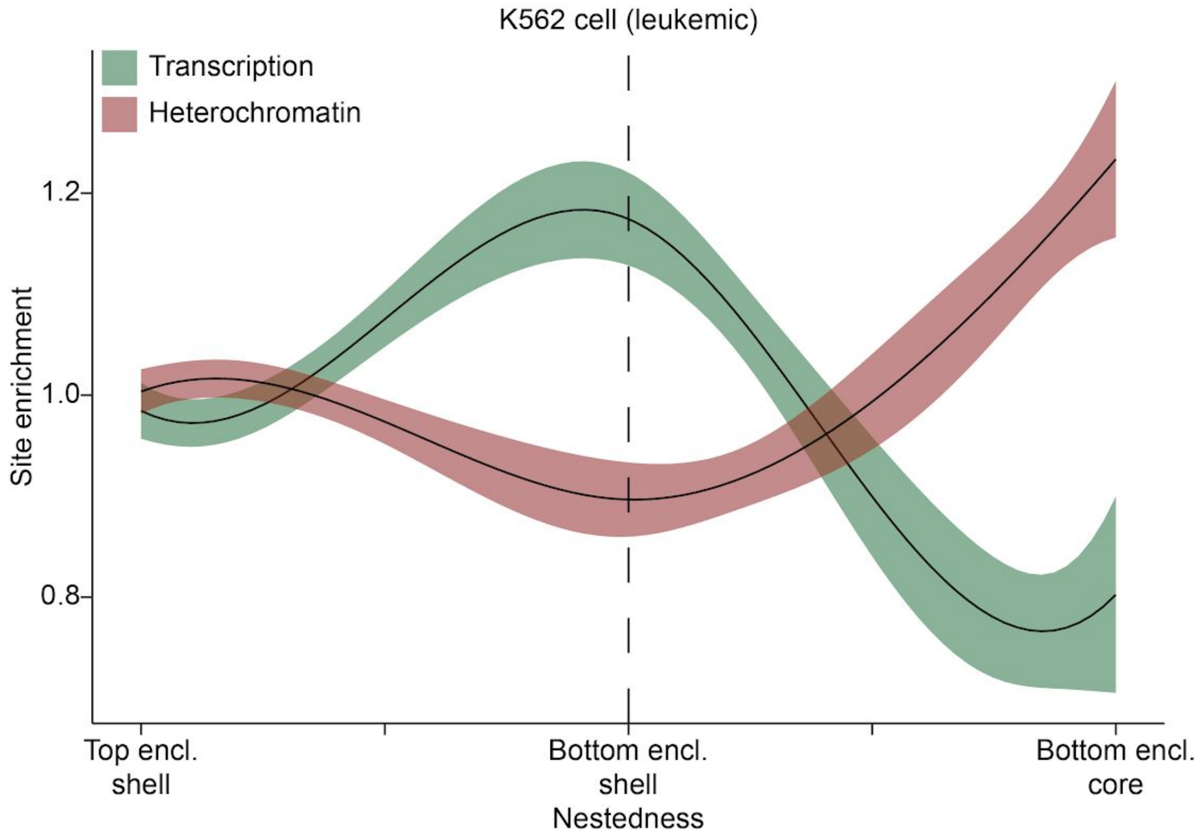
K562 cell (leukemic)
Transcription
Heterochromatin
Site enrichment
1.2
1.0
0.8
Top encl. shell
Bottom encl. shell
Bottom encl. core
Nestedness

Supp. Figure 10: Epigenomic enrichment profile along BPT nestedness for active (green) and inactive (red) epigenomic features for human umbilical vein endothelial cells (HUVEC) (top) and K562 cells (bottom). Transcription trends were derived using the chromHMM mnemonic annotation. Heterochromatin trends were derived using chromHMM mnemonic annotation for HUVEC and ChIP-seq data from (37) for K562. Trend lines correspond to a b-spline smoothing with 4 degrees of freedom and the shaded region indicates their 95% confidence interval.

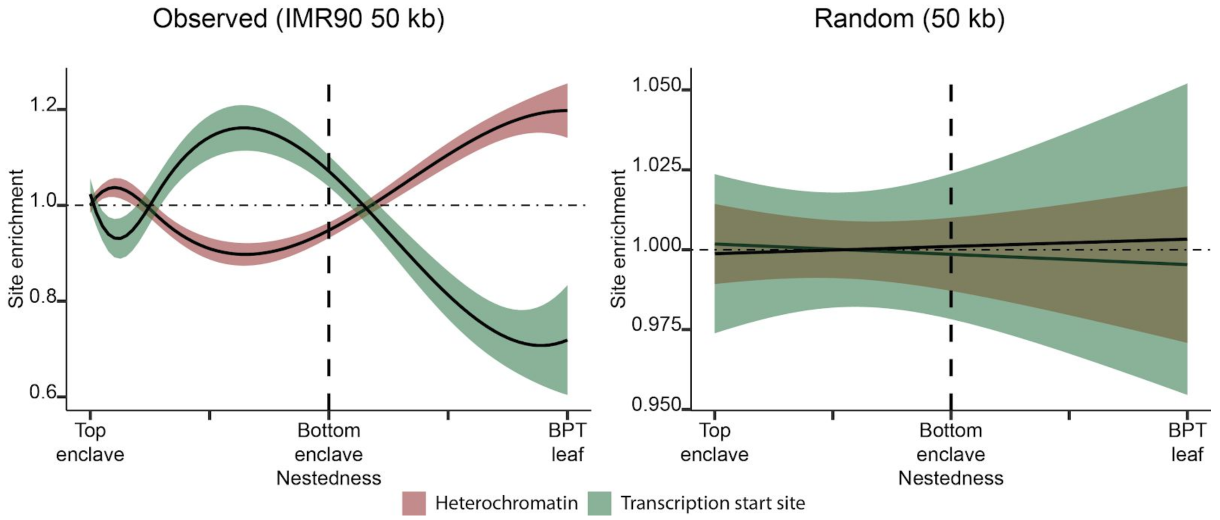

Supp. Figure 11: Epigenomic enrichment profile along BPT nestedness for active (green) and inactive (red) epigenomic features for observed BHi-Cect clustering (IMR90 50 kb) (left) and random clustering (right). Transcription start site trends were derived using the chromHMM mnemonic annotation. Heterochromatin trends were also derived using chromHMM mnemonic annotation. Trend lines correspond to a b-spline smoothing with 4 degrees of freedom and the shaded region indicates their 95% confidence interval.

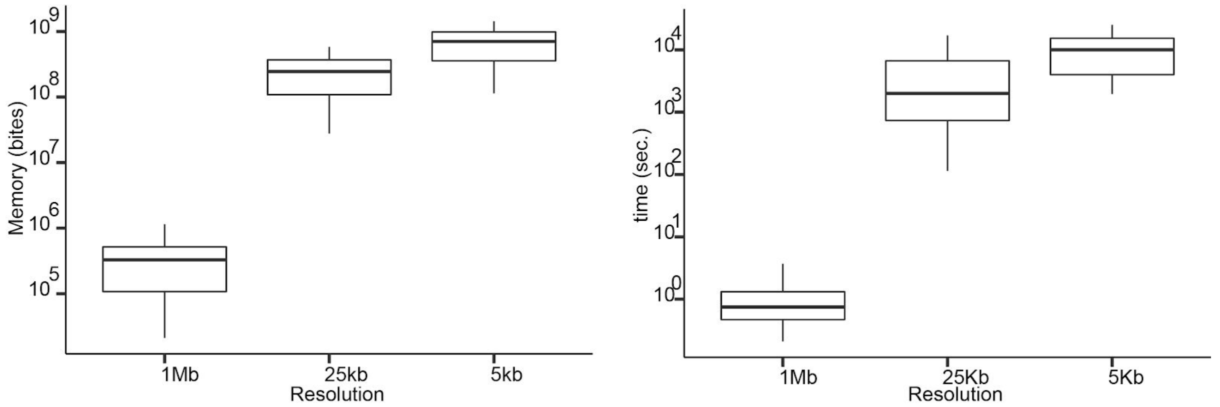

Supp. Figure 12: Memory and time benchmarks when analyzing different resolution Hi-C data (5 kb, 25 kb and 1 Mb). Boxplot reporting for every chromosome at the considered resolution the time and memory usage required to run BHi-Cect. We observe an increase in order of magnitude regarding both time taken (<1 sec for 1 Mb to 2-3 hours for 5 kb) and memory required (100's kb for 1 Mb to >1 Gb for 5 kb) with higher resolution data. We ran BHi-Cect on a desktop with the following specifications: 32 gigabyte. RAM, Intel Core i7-6700 CPU 3.4 GHz.

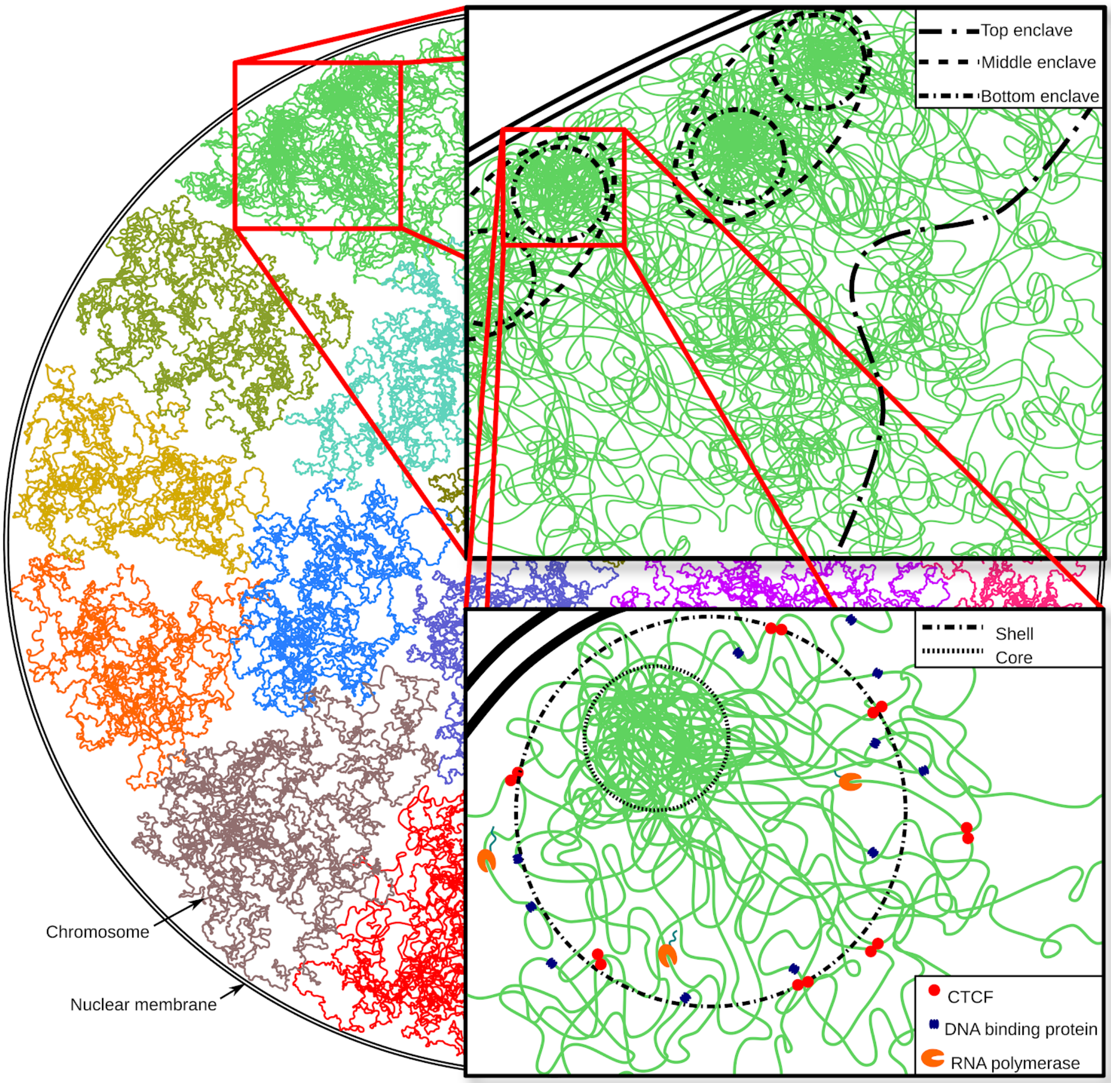

Supp. Figure 13: Proposed interpretation of the multi-scale hierarchical architecture of chromosomes. The genome is organised into "enclaves" that display a series of non-contiguous, interwoven and nested clusters. Transcriptomic and epigenomic functions from various molecular factors are significantly correlated with the DNA nestedness level within the enclave. Particularly, the boundary loci of bottom enclaves tend to coincide with numerous DNA binding and remodelling factors including active RNA polymerase II and CTCFs while the most nested regions tend to be enriched in heterochromatin with reduced accessibility.
